# Elevated vesicular Zn^2+^ in dorsal root ganglion neurons expressing the transporter TMEM163 causes age-associated itchy skin in mice

**DOI:** 10.1371/journal.pbio.3002888

**Published:** 2024-11-27

**Authors:** Fang Tong, Shuai Liu, Chen Zhang, Xicheng Gu, Huan Yang, Bin Zhou, Yun-Yun Wang, Jianwei Chen, Qianhui Qu, Ye Gong, Haili Pan, Chen Liang, Changlin Li, Xin Zhang, Qingjian Han

**Affiliations:** 1 Shanghai Stomatological Hospital & School of Stomatology, State Key Laboratory of Medical Neurobiology and MOE Frontiers Center for Brain Science, Institutes of Brain Science, Institutes of Biomedical Sciences, Fudan University, Shanghai, China; 2 Neurological Institute of Jiangxi Province and Department of Neurology, Jiangxi Provincial People’s Hospital, The First Affiliated Hospital of Nanchang Medical College, Nanchang, China; 3 Guangdong Institute of Intelligence Science and Technology, Hengqin, Zhuhai, China; 4 The Affiliated Wuxi People’s Hospital of Nanjing Medical University, Wuxi People’s Hospital, Wuxi Medical Center, Nanjing Medical University, Nanjing, China; Columbia University Irving Medical Center, UNITED STATES OF AMERICA

## Abstract

The prevalent itching condition associated with aging, historically referred to as senile pruritus, diminishes quality of life. Despite its impact, effective treatments remain elusive, largely due to an incomplete understanding of its pathological cause. In this study, we reveal a subset of dorsal root ganglion neurons enriched with Zn^2+^ that express the vesicular Zn^2+^ transporter TMEM163. These neurons form direct synapses with and modulate the activity of spinal NPY^+^ inhibitory interneurons. In aged mice, both the expression of TMEM163 and the concentration of vesicular Zn^2+^ within the central terminals of TMEM163^+^ primary afferents show marked elevation. Importantly, the excessive release of vesicular Zn^2+^ significantly dampens the activity of NPY^+^ neurons, triggering the disinhibition of itch-transmitting neural circuits and resulting in chronic itch. Intriguingly, chelating Zn^2+^ within the spinal dorsal horn effectively relieves itch in aged mice. Our study thus unveils a novel molecular mechanism underlying senile pruritus.

## Abstract 2

What causes an itchy skin in old age? This study using aged mice shows that elevated release of vesicular Zn2+ from TMEM163-positive neurons dampens the activity of NPY neurons, thereby triggering the disinhibition of itch-transmitting neural circuits, leading to chronic itch in aging.

## Introduction

Itch represents a distressing sensation and emotional experience that provokes scratching or a strong desire to scratch. Intractable itch, a prevalent skin-related complaint among elderly individuals, significantly decreases their overall quality of life. In the spinal dorsal horn, several subpopulations of excitatory interneurons including gastrin-releasing peptide receptor (GRPR)^+^ interneurons [1,2], Urocortin 3 (UNC3)^+^ interneurons (INs) [3], Neuropeptide Y receptor type 1 (NPY1R)^+^ interneurons [4], and Tachykinin 2 (TAC2)^+^ interneurons [5] have been identified as critical regulator of itch transmission. Recent studies have uncovered a distinct subpopulation of neuropeptide Y (NPY)-positive INs within the spinal cord, which receive input from low-threshold mechanoreceptors (LTMRs) in hairy skin and tightly gate the transmission of itch, and the dysfunction of LTMRs-NPY^+^ INs can cause the pathogenesis of skin lesion and chronic itch [3,6,7]. However, the identity of the LTMRs connected to NPY^+^ INs and the molecular mechanisms underlying the loss of inhibitory function in NPY^+^ neurons during chronic itch remains elusive.

Zinc (Zn), the second most abundant trace element in the body, serves dual roles as a structural component of proteins and a signaling molecule regulating various vital processes [8]. The majority of Zn in neurons exists as free Zn^2+^ stored in synaptic vesicles [8]. Vesicular Zn^2+^ is released into the synaptic cleft together with glutamate during neuronal firing, modulating synaptic plasticity and neural transmission [9,10]. Mice lacking vesicular Zn^2+^ exhibit deficits in various processes, including context recognition, learned fear, extinction, spatial working memory, and cognition [9,11–14]. Two distinct classes of Zn^2+^ transporters exist: Zrt- and Irt-related proteins (ZIPs), which facilitate the movement of Zn^2+^ from extracellular or intracellular organelle compartments into the cytosol, and zinc transporters (ZnTs), which mediate Zn^2+^ transport in the opposite direction [8]. To date, a total of 14 ZIPs and 11 ZnTs have been identified. Among them, TMEM163 (also known as SV31 or ZnT11) was initially characterized as a synaptic vesicle membrane protein and is primarily localized to the vesicular membrane, where it plays a crucial role in the transportation of Zn^2+^ from the cytosol into synaptic vesicles [15–17]. NMDA receptors (NMDARs) are one of the key targets of Zn^2+^ [18–20]; Zn^2+^ binds to NR2A when it is present at low levels (nM) [18] and to NR2B when it is present at high levels (μm) to inhibit NMDARs [21]. Mice harboring mutations in the Zn^2+^-binding sites of NR2A were found to exhibit increased heat hyperalgesia and mechanical allodynia in both inflammatory pain and neuropathic pain mouse models. Moreover, the analgesic effect of Zn^2+^ is abolished in these mice, indicating that the Zn^2+^ and NR2A interaction is essential for the analgesic properties of Zn^2+^ [22]. AMPA receptors (AMPARs) play a critical role in fast excitatory glutamatergic neurotransmission, which can also be inhibited by Zn^2+^ released from synaptic vesicles [23]. Zn^2+^ released from dorsal cochlear nucleus (DCN), boutons synapsing onto CA1 neurons, or mossy fibers synapsing onto CA3 neurons inhibits AMPARs [24–27].

In this study, we found that there is one subpopulation of DRG neurons that express the vesicular Zn^2+^ transporter TMEM163. These neurons can form monosynaptic connections with spinal NPY^+^-derived inhibitory INs and modulate their neuronal activity by releasing Zn^2+^. During aging, both the expression of TMEM163 and the concentration of vesicular Zn^2+^ stored in the central terminals of TMEM163^+^ DRG neurons significantly increased. Stimulation increased Zn^2+^ release into the synaptic cleft, inhibiting the activity of NPY^+^ INs, leading to disinhibition of itch-transmitting neural circuitry and chronic itch. Targeting primary sensory neuron-derived Zn^2+^ in the spinal dorsal horn effectively alleviates chronic itch in aged mice.

## Results

### Zn^2+^ transport from DRG to spinal dorsal horn increases during aging

**Fig 1:**
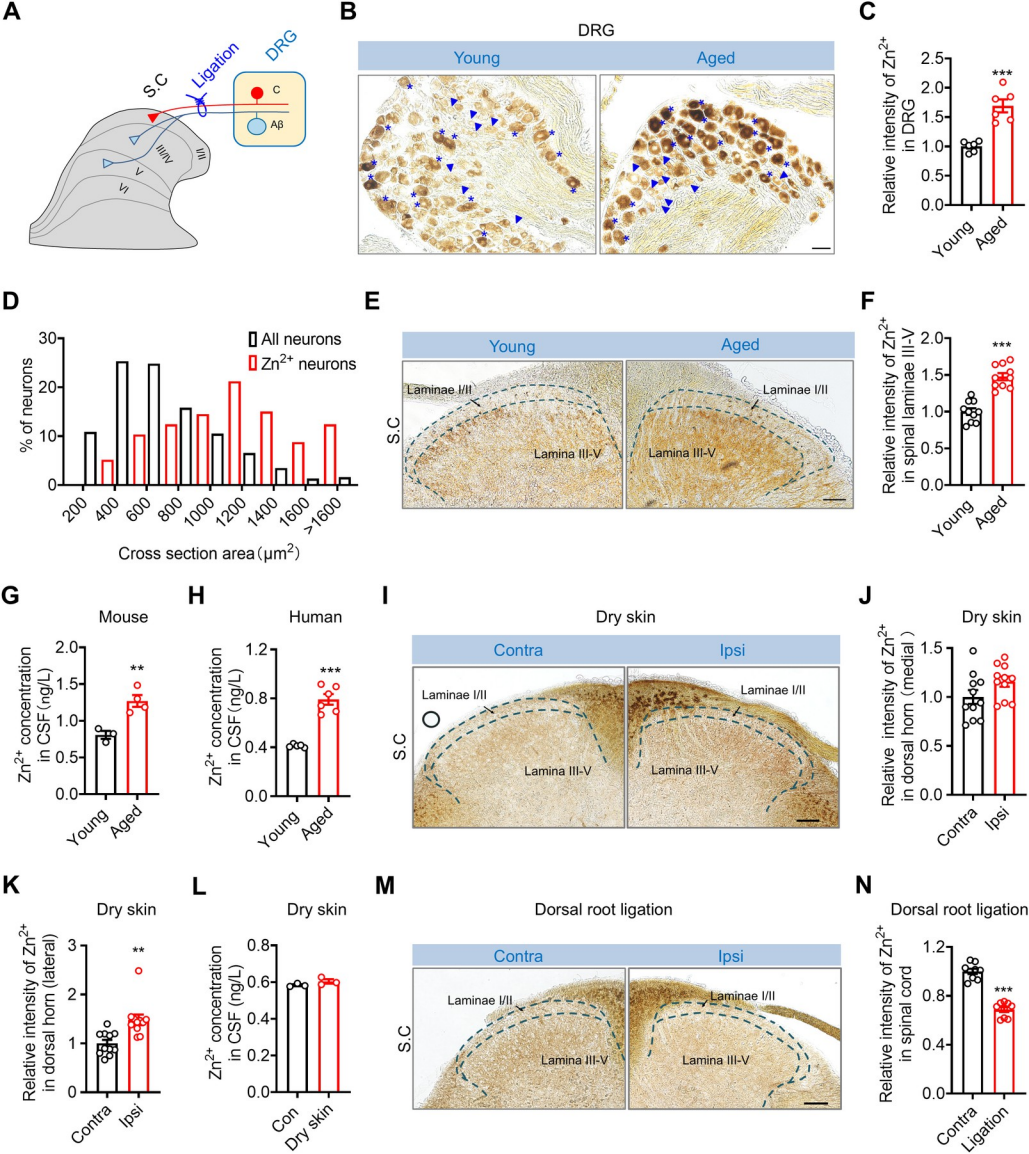
The distribution of vesicular Zn^2+^ in the DRG and spinal cord. (**A**) Schematic diagram illustrating the projection of central branches arising from different DRG neuron subtypes within the spinal dorsal horn and the dorsal root ligation model. (**B**, **C**) ZnS^AMG^ staining in the DRG in young mice and aged mice. (B) Representative images displaying ZnS^AMG^ staining in DRG sections. Scale bar = 50 μm. (C) Quantitative analysis of the mean gray value of ZnS^AMG^. Welch’s *t* test, *n* = 6/group. (**D**) Size distribution of ZnS^AMG^-positive DRG neurons and total DRG neurons in naïve young mice. (**E**, **F**) ZnS^AMG^ staining in the spinal cords of young mice and aged mice. (E) Representative image of ZnS^AMG^ staining in the spinal cord. Scale bar = 50 μm. (F) Quantitative analysis of the mean intensity of ZnS^AMG^ staining; unpaired *t* test, *n* = 10/group. (**G**) The [Zn^2+^] in the CSF of young mice and aged mice; unpaired *t* test, *n* = 8 mice/group. (**H**) The [Zn^2+^] in the CSF of young patients and elderly patients; Welch’s *t* test, *n* = 5–6/group. **(I–K)** ZnS^AMG^ staining in the spinal cords of dry skin mice. (I) Representative image of ZnS^AMG^ staining in the spinal cord. Scale bar = 50 μm. (J, K) Quantitative analysis of the mean intensity of ZnS^AMG^ staining on the contralateral and ipsilateral sides; (J) lateral spinal cord, Mann–Whitney test, *n* = 11/group; (K) medial spinal cord, unpaired *t* test, *n* = 11/group. **(L)** The [Zn^2+^] in the CSF of naïve mice and dry skin model mice; unpaired *t* test, *n* = 6 mice/group. (**M, N**) ZnS^AMG^ staining in the spinal cords of dorsal root ligation model mice. (M) Representative image of ZnS^AMG^ staining in the spinal cord. Scale bar = 50 μm. (N) Quantitative analysis of the mean ZnS^AMG^ staining intensity in spinal cords of dorsal root ligation model mice; unpaired *t* test, *n* = 9/group. All data are expressed as the mean ± SEM. Significant differences were analyzed using unpaired *t* tests; *p < 0.05, **p < 0.01, and ***p < 0.001. The underlying data for Fig 1C, 1D, 1F, 1G, 1H, 1J, 1K, 1L, and 1N can be found in S1 Data. CSF, cerebrospinal fluid; DRG, dorsal root ganglia.

To investigate the role of endogenous Zn^2+^ in itch sensation, we assessed its distribution in the DRG and spinal cord using zinc sulfide autometallography (ZnS^AMG^) staining, which is used to specifically detect free Zn^2+^ [28,29]. The results showed that there was a large amount of Zn^2+^ selectively distributed in large-sized DRG neurons (cross-sectional area ≥600 μm^2^), the central branches of which mainly terminate in laminae III-V of the spinal dorsal horn (Fig 1A–1D). Notably, there was a significant increase in the intensity of ZnS^AMG^ staining specifically within large-sized DRG neurons in aged mice (≥18 months) (Fig 1B and 1C). In the spinal cord, Zn^2+^ was mostly distributed in the deep dorsal horn from laminae III to laminae V (Fig 1E); this area overlapped with the area where the central branches of large-sized DRG neurons terminated. Consistently, the intensity of ZnS^AMG^ staining in both laminae III-V of the spinal dorsal horn and the epidermis of nape region significantly increased in aged mice (Figs 1E and 1F and S1A and S1B). We measured the concentration of Zn^2+^ ([Zn^2+^]) in the cerebrospinal fluid (CSF), and the results showed that the [Zn^2+^] increased from 0.808 ± 0.054 ng/L in young mice (6 to 8 weeks) to 1.270 ± 0.080 ng/L in aged mice (Fig 1G). Consistently, the [Zn^2+^] in the CSF of elderly people (≥54 years) was significantly higher than that in the CSF of young people (≤38 years) (Fig 1H). Dry skin is a common skin condition in older adults [30,31]. Thus, we evaluated the distribution of vesicular Zn^2+^ in the spinal cord and the [Zn^2+^] in the CSF in a well-established acetone–ether–water (AEW) model that recapitulates dry skin observed in elderly patients [30]. The intensity of ZnS^AMG^ staining in the lateral part of dorsal horn, receiving sensory information from nape region, significantly increased in ipsilateral side, while that of medial part was not significantly changed (Fig 1I–1K). However, the [Zn^2+^] in the CSF of dry skin model mice was comparable to that of naïve control mice (Fig 1L), possibly because the pathological changes in the nape are insufficient to elicit the change of [Zn^2+^] in CSF. The selective distribution of Zn^2+^ in large-sized DRG neurons and laminae III-V of the spinal dorsal horn indicated that Zn^2+^ in the deep laminae of the dorsal horn may be transported from large-sized DRG neurons. To test this hypothesis, we performed a spinal dorsal root ligation assay (Fig 1A). Interestingly, our results demonstrated a substantial decrease in the intensity of ZnS^AMG^ staining specifically in laminae III-V on the ipsilateral side compared to the contralateral side 3 days after ligation (Fig 1M and 1N). These findings strongly suggest that Zn^2+^ in the deep laminae of the dorsal horn is indeed transported from DRG neurons and its level increased during aging and in dry skin.

### Zn^2+^ in spinal cord regulates aging-related itch

**Fig 2:**
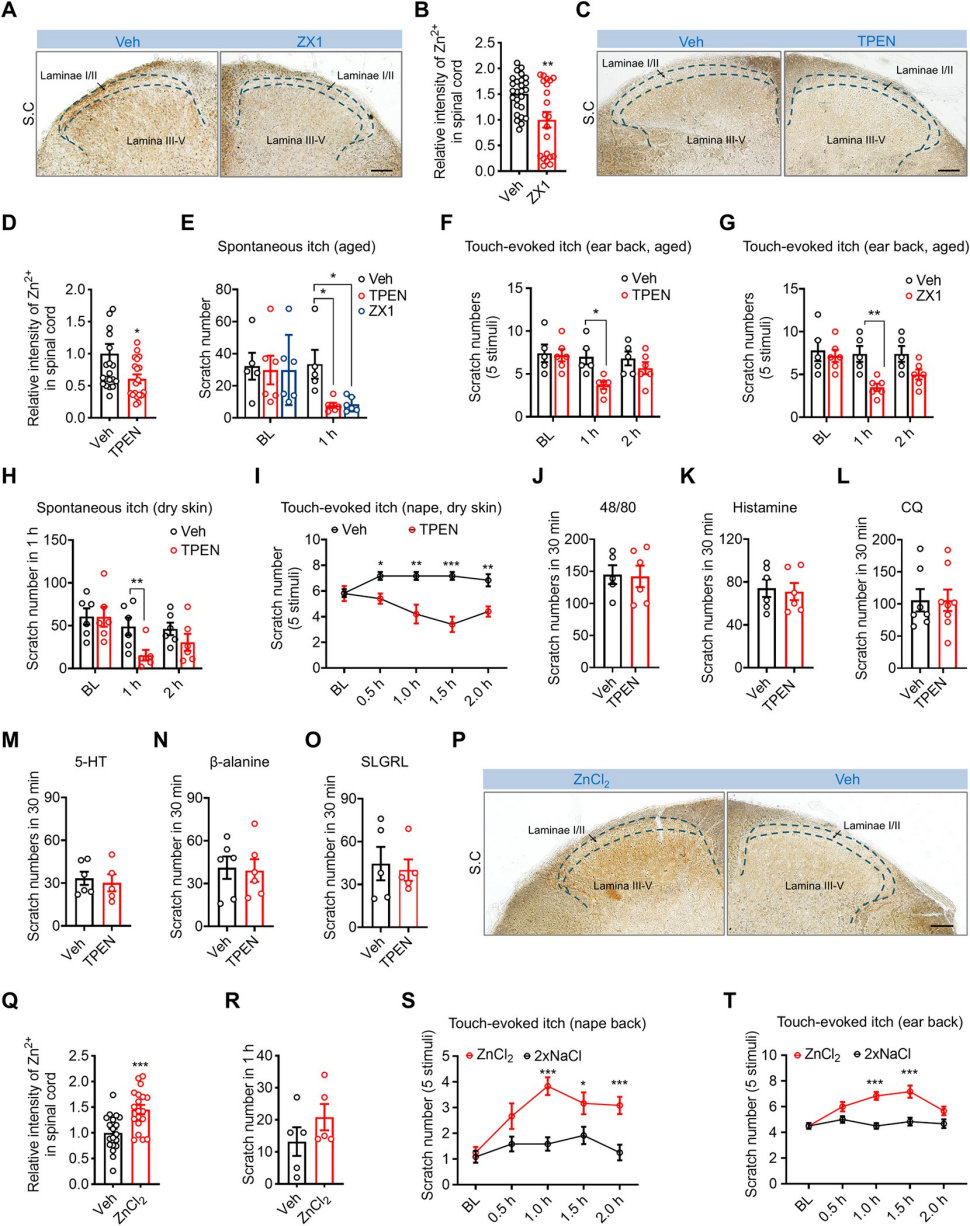
Spinal Zn^2+^ regulates aging-related itch but not acute chemical itch. **(A, B)** ZnS^AMG^ staining in the spinal cords after I.T. injection ZX1. (A) Representative image of ZnS^AMG^ staining in the spinal cord. Scale bar = 50 μm. (B) Quantitative analysis of the mean ZnS^AMG^ staining intensity in spinal cords after I.T. injection ZX1; unpaired *t* test, *n* = 26-21/group. **(C, D)** ZnS^AMG^ staining in the spinal cords after I.T. injection TPEN. (C) Representative image of ZnS^AMG^ staining in the spinal cord. Scale bar = 50 μm. (D) Quantitative analysis of the mean ZnS^AMG^ staining intensity in spinal cords after I.T. injection TPEN; unpaired *t* test, *n* = 21~19/group. (**E**) Spontaneous itch response of aged mice after intrathecal injection of the Zn^2+^ chelators TPEN and ZX1. Two-way ANOVA followed by Sidak’s multiple comparisons test; *n* = 6 mice/group. (**F, G**) Touch-evoked itch response of aged mice elicited with a 0.07 g von Frey filament after intrathecal injection of vehicle, the Zn^2+^ chelator TPEN (100 μm, 10 μl) (F) or ZX1 (100 μm, 10 μl) (G). Two-way ANOVA followed by Sidak’s multiple comparisons test; *n* = 5–6 mice/group. (**H**) Spontaneous itch response of dry skin model mice after intrathecal injection of vehicle or the Zn^2+^ chelator TPEN. Two-way ANOVA followed by Sidak’s multiple comparisons test; *n* = 6 mice/group. (**I**) Touch-evoked itch response of dry skin model mice after intrathecal injection of vehicle or the Zn^2+^ chelator TPEN. Two-way ANOVA followed by Sidak’s multiple comparisons test; *n* = 5–6 mice/group. (**J–O**) Effect of intrathecal injection of vehicle or the Zn^2+^ chelator TPEN on acute chemical itch elicited by intradermal injection of 48/80 (2 μg/μl, 50 μl), histamine (10 μg/μl, 50 μl), CQ (4 μg/μl, 50 μl), 5-HT (6 μg/μl, 50 μl), β-alanine (6 μg/μl, 50 μl), and SLGRL (2 μg/μl, 50 μl). Unpaired *t* test; *n* = 5–6 mice/group. (**P, Q**) ZnS^AMG^ staining in the spinal cords after I.T. injection Vehicle and ZnCl_2_. (P) Representative image of ZnS^AMG^ staining in the spinal cord. Scale bar = 50 μm. (Q) Quantitative analysis of the mean ZnS^AMG^ staining intensity in spinal cords after I.T. injection Vehicle and ZnCl_2_; unpaired *t* test, *n* = 19/group. (**R**) Spontaneous itch response of WT mice after intrathecal injection Vehicle and ZnCl_2_. Unpaired *t* test, *n* = 5 mice/group. (**S, T**) Itch response evoked by touch stimulation of the nape (S) and back of the ear (T) after intrathecal injection of 2 × NaCl and ZnCl_2_. Two-way ANOVA followed by Sidak’s multiple comparisons test, *n* = 6–12 mice/group. All data are expressed as the mean ± SEM; *p < 0.05, **p < 0.01, ***p < 0.001. The underlying data for Fig 2B, 2D, 2E, 2F, 2G, 2H, 2I, 2J, 2K, 2L, 2M, 2N, 2O, 2Q, 2R, 2S, and 2T can be found in S1 Data. SEM, standard error of the mean.

Next, we tested whether spinal Zn^2+^ mediates aging-related itch, including spontaneous itch and mechanical itch hypersensitivity. Mechanical itch hypersensitivity, known as alloknesis, represents an abnormal sensory state in which mechanical stimuli, typically non-itch-inducing, induce intense itchiness, a phenomenon commonly observed in aged skin [32,33]. Aged mice developed both spontaneous itch and mechanical itch hypersensitivity (S2A–S2D Fig), which was consistent with previous studies [3,30]. Intrathecal (*i*.*t*.) injection of the specific Zn^2+^ chelator TPEN (100 μm, 10 μl) or ZX1 (100 μm, 10 μl) [34,35] both can significantly reduce the intensity of ZnS^AMG^ staining in laminae III-V of spinal dorsal horn (Fig 2A–2D). Importantly, these 2 chelators effectively alleviated both spontaneous itch and touch-evoked itch in aged mice in the first hour after injection (Fig 2E–2G). Consistently, both spontaneous itch and touch-evoked itch in the AEW treatment-induced dry skin model mice were effectively alleviated after chelation of spinal Zn^2+^ through *i*.*t*. injection of TPEN (Fig 2H and 2I). However, neither histamine-dependent acute chemical itch (elicited by intradermal injection of compound 48/80 and histamine) nor histamine-independent acute chemical itch (elicited by intradermal injection of chloroquine, serotonin, β-alanine, and SLGRL) were affected by chelation of spinal Zn^2+^ with *i*.*t*. injection of TPEN (Fig 2J–2O). Next, we investigated whether Zn^2+^ can directly elicit itch. The results demonstrated that *i*.*t*. injection of ZnCl_2_ increased the intensity of the Zn^2+^ signal in the spinal dorsal horn (Fig 2P and 2Q). Behavioral tests showed that *i*.*t*. injection of ZnCl_2_ was insufficient to elicit spontaneous itch but significantly potentiated touch-evoked itch stimulated by application of a 0.07 g filament to both the nape and back of the ear (Fig 2R–2T). Collectively, these data indicate that endogenous Zn^2+^ in the spinal cord regulates aging-related itch.

Previous studies have highlighted the essential role of skin Zn^2+^ in both acute itch and chronic itch [36]. We then investigated whether skin Zn^2+^ is involved in aging-associated itch. Our results showed that chelating skin Zn^2+^ via intradermal injection of ZX1 at the ear back significantly reduced both spontaneous and touch-evoked itch in aged mice (S2E and S2F Fig), suggesting that skin Zn^2+^ plays a role in age-associated itch. *I*.*t*. injection is a well-established method for delivering drugs, siRNAs, or AAVs to DRG neurons and SC, as previously described [37–40]. Our findings demonstrated that *I*.*t*. injection of TPEN elicited an obvious tendency of reduction of [Zn^2+^], although this difference was not statistically significant (S2G and S2H Fig), indicating that Zn^2+^ in DRG neurons may be released into the skin and contribute to Zn^2+^ levels. However, *I*.*t*. injection of ZnCl_2_ had no effect on the [Zn^2+^] in the skin (S2I and S2J Fig). These data suggest that *I*.*t*. injection of TPEN-elicited reduction of age-associated itch is partially due to the chelation of skin Zn^2+^.

### TMEM163 mediates Zn^2+^ accumulation in spinal dorsal horn and aging-related itch

**Fig 3:**
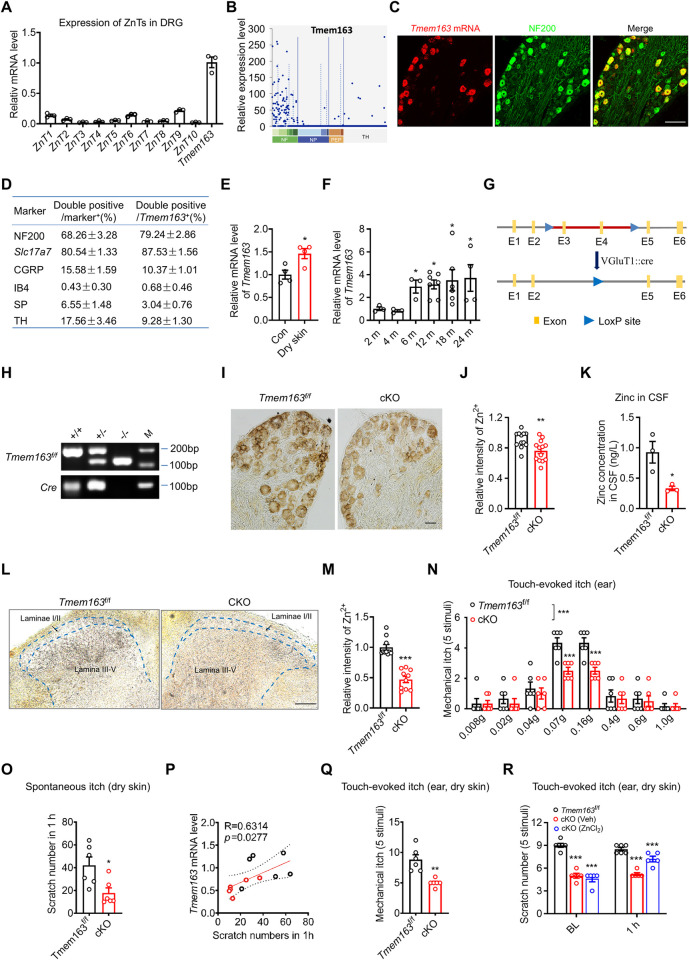
TMEM163 mediates vesicular Zn^2+^ transport and aging-related itch. (**A**) Relative mRNA levels of ZnT1-ZnT10 and TMEM163 in the mouse DRG; *n* = 3/group. The underlying primer data for Fig 3A can be found in S1 Table. (**B**) Profiling of the cell type expressing TMEM163 in the mouse DRG based on a previously published single-cell RNA-seq database (GSE59739). (**C**) Representative images of ISH combined with IHC using a probe targeting mouse *Tmem163* (red) and a large DRG neuron marker (NF3200). Scale bar = 100 μm. (**D**) Profiling of the cell type expressing TMEM163 in the DRG. Immunofluorescence staining using markers for different DRG neuron subtypes (large-sized DRG neurons (NF200), peptidergic DRG neurons (SP and CGRP), nonpeptidergic DRG neurons (IB4), and C-LTMRs (TH)) was performed; *n* = 17 /group. (**E**) Relative mRNA level of *Tmem163* in the DRG of naïve control mice and dry skin model mice; unpaired *t* test, *n* = 4 /group. (**F**) Relative mRNA level of Tmem163 in the mouse DRG at 2 months (2 m), 4 months (4 m), 6 months (6 m), 12 months (12 m), 18 months (18 m), and 24 months (24 m). Unpaired *t* test; *n* = 3–7/group. (**G**) Schematic diagram of the strategy employed to generate *VGlut1*::*cre;Tmem163*^f/f^ mice. (**H**) Genotyping of *Tmem163* cKO mice using the primer listed in the S1 Table. The gel image shows the bands corresponding to WT mice (132 bp), *Tmem163*^*f/f*^ mice (202 bp), and Cre recombinase (100 bp). M indicates molecular weight marker bands for reference. (**I, J**) ZnS^AMG^ staining in the DRG of WT mice and *Tmem163* cKO mice. (I) Representative images of ZnS^AMG^ staining in DRG sections. Scale bar = 20 μm. (J) Quantitative analysis of the mean intensity of ZnS^AMG^ staining, Mann–Whitney test, *n* = 13–14/group. (**K**) Quantification of the [Zn^2+^] in the CSF of WT mice and Tmem163 cKO mice; *n* = 3/group. (**L, M**) ZnS^AMG^ staining in the spinal cords of WT mice and *Tmem163* cKO mice. (L) Representative images of ZnS^AMG^ staining in spinal cord sections. Scale bar = 50 μm. (M) Quantitative analysis of the mean intensity of ZnS^AMG^ staining, Mann–Whitney test, *n* = 10/group. (**N**) Touch-evoked itch response elicited by application with different von Frey filaments in *Tmem163*^f/f^ mice and *Tmem163* cKO mice; two-way ANOVA followed by Sidak’s multiple comparisons test; *n* = 6 /group. (**O**) Spontaneous itch response of *Tmem163*^f/f^ and *Tmem163* cKO mice under dry skin conditions. Unpaired *t* test; *n* = 6/group. (**P**) Correlation analysis of *Tmem163* mRNA level with spontaneous itch number of *Tmem163*^f/f^ mice and *Tmem163* cKO mice. Pearson correlation analyze; *n* = 6/group. (**Q**) Touch-evoked itch response of *Tmem*163^f/f^ and *Tmem163* cKO mice under dry skin conditions. Welch’s *t* test; *n* = 6/group. (**R**) Touch-evoked itch response of *Tmem163*^f/f^ and *Tmem163* cKO mice after intrathecal injection of vehicle or ZnCl_2_. Two-way ANOVA followed by Sidak’s multiple comparisons test; *n* = 5–6/group. All data are expressed as the mean ± SEM; *p < 0.05, **p < 0.01, ***p < 0.001. The underlying data for Fig 3A, 3D, 3E, 3F, 3J, 3K, 3M, 3N, 3O, 3P, 3Q, and 3R can be found in S1 Data. cKO, conditional knockout; CSF, cerebrospinal fluid; DRG, dorsal root ganglia; ISH, in situ hybridization; SEM, standard error of the mean; WT, wild-type.

To elucidate the molecular mechanism underlying the accumulation of spinal Zn^2+^ in deep laminae of spinal dorsal horn and the selective regulatory effect of spinal Zn^2+^ on aging-related itch, we conducted a comprehensive analysis of Zn^2+^ transporters (ZnTs) responsible for transporting Zn^2+^ from the cytosol into organelles [8] within the DRG. Our results revealed that the expression level of TMEM163 (ZnT11) in the DRG was markedly higher than that of other ZnTs (Fig 3A). Furthermore, we examined the expression profiles of ZnTs in a single-cell sequencing database [41,42], and the results showed that TMEM163, but not ZnT1-10, was selectively highly expressed in large-sized DRG neurons (neurofilament (NF)-containing DRG neurons) (Figs 3B and S3A), which was consistent with the observed distribution of Zn^2+^ in DRG (Fig 1B and 1D). Consistently, ISH combined with IHC showed that *Tmem163* was mainly expressed in large-sized DRG neurons, with 79.24 ± 2.86% of *Tmem163*^+^ DRG neurons expressing NF200 and 87.53 ± 1.56% of *Tmem163*^+^ DRG neurons being positive for *Slc17a7* (VGluT1) (Figs 3C, 3D, S3B, and S3C). In mice with dry skin and aged mice, the expression of *Tmem163* increased significantly (Fig 3E and 3F), indicating the potential role of this transporter in aging-related itch. To test the role of *Tmem163* in aging-related Zn^2+^ accumulation in the spinal dorsal horn and aging-related itch, we generated *Tmem163* conditional knockout (cKO) mice in which *Tmem163* was selectively deleted in VGluT1^+^ DRG neurons (Fig 3G and 3H). In the cKO mice, the intensity of ZnS^AMG^ staining in the DRG was significantly reduced (Fig 3I and 3J). Furthermore, the concentration of Zn^2+^ in the CSF was markedly decreased from 0.927 ± 0.177 ng/L in WT mice to 0.332 ± 0.035 ng/L in cKO mice (Fig 3K). We also examined the distribution of Zn^2+^ in the deep laminae of the spinal dorsal horn in cKO mice and found a sharp reduction in the intensity of ZnS^AMG^ staining (Fig 3L and 3M). These findings collectively indicate the critical role of TMEM163 in the accumulation of Zn^2+^ in large-sized DRG neurons and the deep laminae of the spinal dorsal horn. We also assessed the itch behavior of the cKO mice under naïve conditions and in the context of dry skin. Touch-evoked itch was induced by stimulation of the ear region with graded von Frey filaments, and the results showed that itch evoked by the filaments with bending forces of 0.07 g and 0.16 g was significantly decreased in the cKO mice under naïve conditions (Fig 3N). Similarly, touch-evoked itch elicited by application of Von Frey filaments to the nape region was also reduced (S3D Fig). Under dry skin conditions, both spontaneous itch and touch-evoked itch were decreased dramatically in cKO mice (Figs 3O–3Q and S3E). Importantly, the reduction in touch-evoked itch can be reversed by *i*.*t*. injection of ZnCl_2_ under dry skin conditions (Fig 3R). Collectively, these findings provide compelling evidence that the Zn^2+^ transporter TMEM163 mediates the accumulation of Zn^2+^ in large-sized DRG neurons and the deep laminae of the spinal dorsal horn, thereby playing a vital role in the pathogenesis of aging-related itch.

### TMEM163 and VGluT1 are colocalized in the same presynaptic vesicles

**Fig 4:**
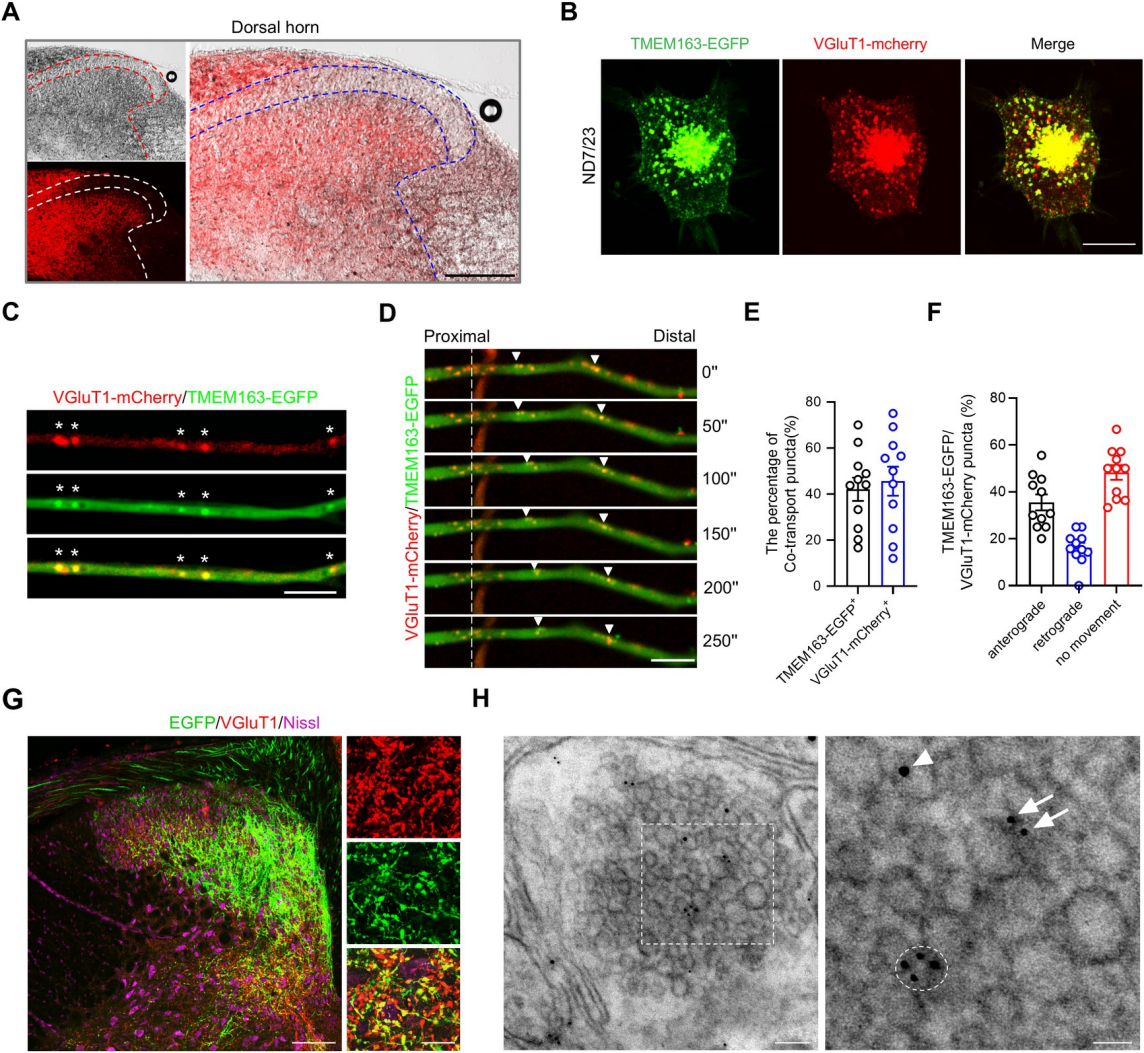
TMEM163 colocalizes with VGluT1 on synaptic vesicles. (**A**) ZnS^AMG^ staining in the spinal cords of VGluT1::Cre;Ai14 mice. Scale bar = 100 μm. (**B**) Overexpression of mouse TMEM163-EGFP (green) and mouse VGluT1-mCherry (red) in ND7/23 cells. Scale bar = 10 μm. (**C, D**) TMEM163-EGFP (green) and VGluT1-mCherry (red) were overexpressed in cultured DRG neurons by electroporation. (C) VGluT1 (red) colocalized with TMEM163 (green) in the axons of cultured DRG neurons. (D) Live-cell imaging reveals the cotransport of TMEM163-EGFP (green) and VGluT1-mCherry (red) in the same vesicle. Scale bar = 5 μm. (**E, F**) Quantitative analysis of VGluT1 (red) cotransport with TMEM163 (green) in the axons of cultured DRG neurons. (E) The percentage of TMEM163-positive puncta containing VGluT1 and the percentage of VGluT1-positive puncta containing TMEM163, *n* = 11. (F) The percentage of VGluT1 and TMEM163 double-positive puncta exhibiting anterograde transport, retrograde transport, and no movement; *n* = 11. (**G**) Immunofluorescence staining of the spinal cord following intrathecal injection of AAV-TMEM163-EGFP into 6- to 8-week-old mice. Spinal cord tissue was harvested and subjected to immunostaining using primary antibodies against VGluT1 (red) and Nissl staining (purple). Scale bars = 100 μm (left), 20 μm (right). (**H**) Immunogold labeling of EGFP (10 nm) and VGluT1 (6 nm) in spinal cord slices from mice infected with AAV-TMEM163-EGFP. The dashed line circle indicates a vesicle containing both TMEM163-EGFP and VGluT1. The arrowhead indicates a TMEM163-EGFP-positive vesicle, and the arrow indicates a VGluT1-positive vesicle. Scale bars = 200 nm (left) and 50 nm (right). The underlying data for Fig 4E and 4F can be found in S1 Data. AAV, adeno-associated virus; DRG, dorsal root ganglia.

Next, we further investigated how vesicular Zn^2+^ is transported from the DRG to the spinal dorsal horn by TMEM163 and released. A previous study showed that Zn^2+^ is mostly stored in the same synaptic vesicles as Glu and released together with Glu into the synaptic cleft during neuronal firing, leading to a transient increase in the free Zn^2+^ concentration in the synaptic cleft, which further modulates synaptic transmission in the hippocampus [8,14,43,44]. VGluTs localized on the membrane of synaptic vesicles play a critical role in incorporating Glu into synaptic vesicles. Because VGluT1 is selectively expressed in large DRG neurons [45], similar to the expression pattern of TMEM163, we examined the colocalization of TMEM163 and VGluT1 at the subcellular level. Data from VGluT1::cre; Ai14 mice showed that the central branches of VGluT1^+^ DRG neurons terminated in the deep laminae of the spinal dorsal horn, overlapping with the ZnS^AMG^ signal (Fig 4A). Then, we induced overexpression of TMEM163-EGFP and VGluT1-mCherry in the ND7/23 cell line, a hybridized cell line consisting of mouse neuroblastoma and rat DRG neurons. TMEM163 colocalized with VGluT1 in vesicle-like punctate structures (Fig 4B). Then, we employed electroporation to induce overexpression of TMEM163-EGFP and VGluT1-mCherry in cultured DRG neurons. Remarkably, we observed distinct colocalization of TMEM163-EGFP and VGluT1-mCherry within the same vesicle-like puncta in the cell body, neurites, and nerve terminals (Fig 4C and 4D). Quantitative analysis showed that 42.10% of TMEM163-EGFP^+^ vesicle-like puncta were vGluT1-mCherry-positive and 45.62% of vGluT1-mCherry^+^ vesicle-like puncta were TMEM163-EGFP-positive (Fig 4E). Live cell imaging was also used to trace the cotransport of TMEM163-EGFP and vGluT1-mCherry. The results showed that 35.51% of double-positive vesicle-like puncta were anterogradely transported toward the nerve terminal, 16.19% of them were retrogradely transported toward the cell body, and 48.30% of them did not show any movement (Fig 4D–4F).

To investigate the potential colocalization of TMEM163 and VGluT1 on presynaptic vesicles, we employed immunoelectron microscopy. Because specific antibodies against TMEM163 are not available, we utilized an adeno-associated virus (AAV) vector expressing TMEM163 fused with an EGFP reporter at its C-terminus (AAV-TMEM163-EGFP). Subsequently, we administered AAV-TMEM163-EGFP (titer: 1.045E+12 vg/ml, 10 μl) by *i*.*t*. injection to specifically infect large DRG neurons, whose central nerve branch fibers predominantly terminated in the deep laminae of the spinal dorsal horn (Fig 4G). Then, we labeled EGFP with 10 nm gold particles and VGluT1 with 6 nm gold particles separately. The results demonstrated the presence of TMEM163-EGFP and VGluT1 either in the same presynaptic vesicles or distributed across separate presynaptic vesicles (Figs 4H, S4A, and S4B). Quantitative analysis showed that 20.24 ± 1.711 (%) 10 nm gold particles colocalized with 6 nm gold particles and 11.33 ± 1.624 (%) 10 nm gold particles colocalized with 10 nm gold particles in the same presynaptic vesicles (S4C Fig). These findings are consistent with the observed localization of TMEM163 and VGluT1 in ND7/23 cells and cultured DRG neurons (Fig 4B–4F). Collectively, these data strongly indicate that Zn^2+^ and Glu may be incorporated into the same vesicles, cotransported to nerve terminals, and subsequently coreleased upon stimulation and depolarization of TMEM163-positive primary afferents.

### TMEM163^*+*^ primary afferents form direct synaptic connections with NPY^+^ INs

**Fig 5:**
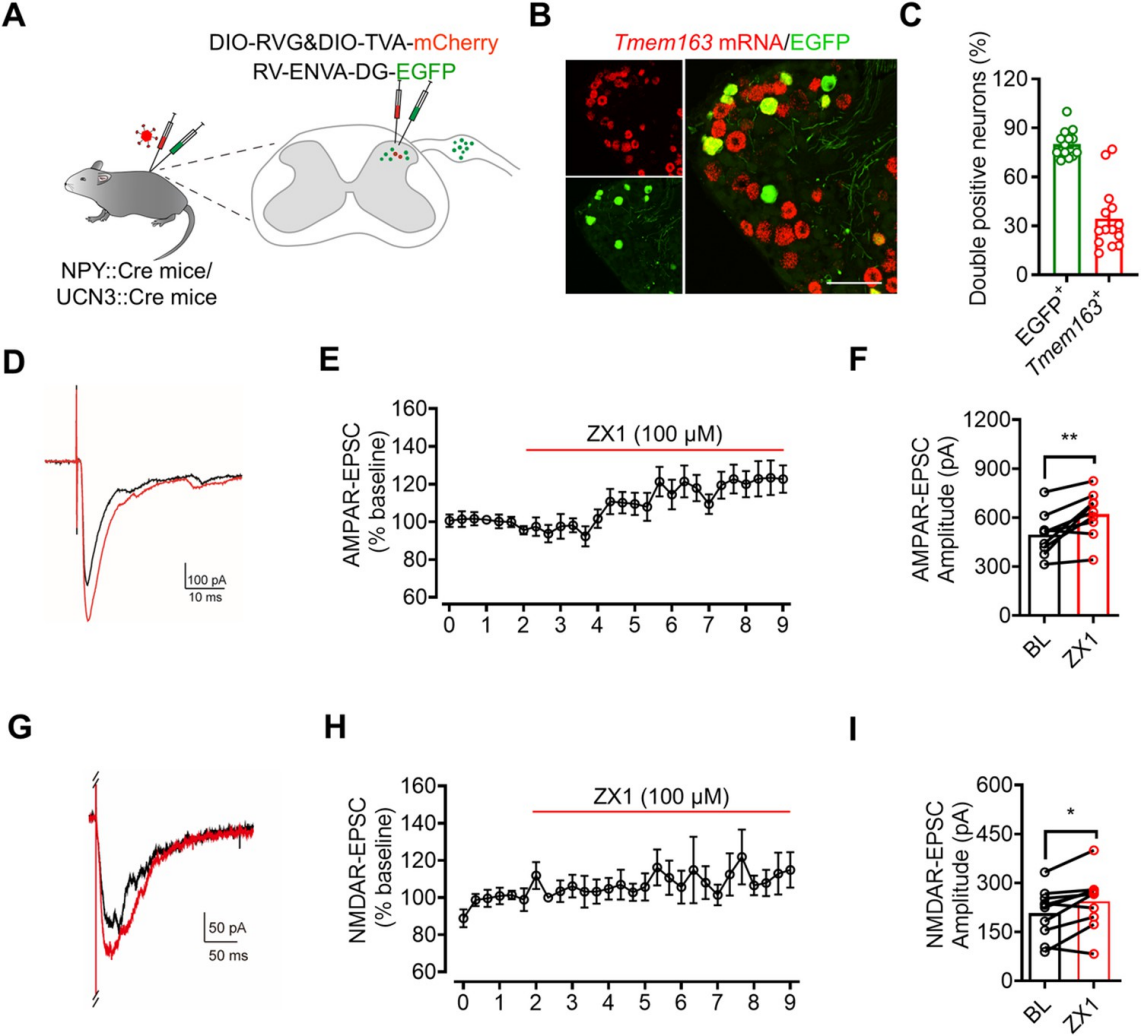
TMEM163^+^ primary afferents form monosynaptic connections with NPY^+^ INs. (**A**) Schematic diagram illustrating the strategy for retrograde transsynaptic tracing of the upstream neurons of NPY::Cre INs or UCN3::Cre INs in the DRG by intraspinal injection of viruses. NPY^+^ mice or UCN3::Cre mice received intraspinal injection of an AAV vector encoding Cre-dependent RVG (AAV2/5-hEF1a-DIO-RVG-WPRE-pA) and TVA fused with mCherry (AAV2/9-CAG-DIO-TVA-mCherry-WPRE-pA) into laminae III-V. After 3 weeks, EnvA-pseudotyped rabies virus (RV-ENVA-DG-EGFP) was injected for retrograde tracing. (**B**) Representative images showing infected presynaptic neurons (EGFP^+^) of NPY^+^ INs in the DRG and their coexpression of *Tmem163* using ISH. Scale bar = 100 μm. (**C**) Quantitative analysis of the data in (B). (**D**) Representative eEPSCs in laminae III-V NPY^+^ INs before and after ZX1 application (hold at −70 mV). (**E**) Time course of the change in eEPSC amplitude before and after ZX1 application; *n* = 9 cells. (**F**) Quantitative analysis of the eEPSC amplitude; paired *t* test; *n* = 9 cells/group. (**G**) Representative eEPSCs in laminae III-V NPY^+^ INs before and after ZX1 application (hold at −40 mV). (**H**) Time course of the change in eEPSC amplitude before and after ZX1 application; *n* = 10 cells. (**I**) Quantitative analysis of the eEPSC amplitude; paired *t* test; *n* = 10 cells/group. All data are expressed as the mean ± SEM; *p < 0.05, **p < 0.01. The underlying data for Fig 5C, 5E, 5F, 5H, and 5I can be found in S1 Data. AAV, adeno-associated virus; DRG, dorsal root ganglia; IN, interneuron; ISH, in situ hybridization; SEM, standard error of the mean.

To unravel the specific involvement of vesicular Zn^2+^ stored in the central terminals of TMEM163^+^ primary afferents in mediating aging-related itch, we investigated the postsynaptic neurons of these primary afferents within the spinal cord. Recognizing the critical role of inhibitory INs derived from the NPY^+^ lineage in itch [3], we aimed to determine whether *Tmem163*^+^ primary afferents directly synapse with NPY^+^ spinal inhibitory INs. In the spinal cord of NPY::cre;Ai3 mice, NPY^+^-derived inhibitory INs, distributed in laminae I-V, were labeled with EGFP (S5A Fig). Given that *Slc17a7* (which encodes VGluT1) mostly colocalized with *Tmem163* in the DRG (Figs 3D and S3C), we assumed that the primary afferents of VGluT1^+^ neurons and *Tmem163*^*+*^ neurons in the spinal cord have the same projections and make the same synaptic connections. There were abundant VGluT1^+^ (*Tmem163*^*+*^) nerve terminals surrounding NPY^+^-derived INs in laminae III-V of the spinal cord (S5B Fig), indicating that there was a possible direct synaptic connection between VGluT1^+^ nerve terminals and NPY^+^-derived INs. To identify the presynaptic neurons in the DRG that formed direct synapses with NPY^+^ INs, we performed retrograde trans-monosynaptic tracing by injecting an AAV vector encoding a Cre-dependent RVG (AAV2/5-hEF1a-DIO-RVG-WPRE-pA) and TVA fused with mCherry (AAV2/9-CAG-DIO-TVA-mCherry-WPRE-pA) into laminae III-V of NPY-*Cre* mice and then injected EnvA-pseudotyped rabies virus (RV-ENVA-DG-EGFP) into the mice 3 weeks later (Fig 5A). The results showed that 38.2% of mCherry^+^ neurons expressing EGFP in the spinal dorsal horn (S6A Fig), and there were EGFP^+^ neurons in the DRG, with 80.09 ± 2.209 (%) of these neurons expressing *Tmem163*, 81.90 ± 4.448 (%) of them expressing the A-fiber-generating DRG neuron marker NF200, only 2.50 ± 2.007 (%) of them expressing the Aδ-LTMR marker *Ntrk2* (TrkB), and none of them expressing the C-LTMR marker tyrosine hydroxylase (TH) (Figs 5B, 5C, S6B, and S6C). The same strategy was used to trace the presynaptic neurons of Ucn3::Cre INs in the DRG. Ucn3::Cre INs are mainly distributed in laminae II-III of spinal dorsal horn (S6D Fig). In contrast, only 27.74 ± 5.007 (%) of EGFP^+^ DRG neurons traced in Ucn3::Cre mice coexpressed *Tmem163* (S6E and S6F Fig). Collectively, these data strongly indicate that NPY^+^-derived INs receive monosynaptic inputs from *Tmem163*^+^ primary afferents, whereas Ucn3::Cre neurons exhibit minimal monosynaptic connections with *Tmem163*^+^ primary afferents.

### Spinal Zn^2+^ modulates the neuronal activity of NPY^+^ neurons via AMPARs and NMDARs

Next, we investigated whether spinal Zn^2+^ stored in the central terminals of *Tmem163*^*+*^ primary afferents modulate the neuronal activity of NPY^+^ INs. Neither the amplitude nor the frequency of mEPSCs was affected by the chelation of Zn^2+^ with TPEN (100 μm) or ZX1 (100 μm) (S7A–S7K Fig). Next, we sought to investigate the impact of spinal Zn^2+^ chelation on the synaptic responses of NPY^+^ inhibitory INs mediated by AMPA receptors (AMPARs) and NMDA receptors (NMDARs). The amplitude of AMPAR-mediated EPSCs (AMPAR-EPSCs) was significantly larger in the presence of ZX1 (Fig 5D–5F). We further examined focally evoked NMDAR-mediated EPSCs (NMDAR-EPSCs) in the presence of NBQX (10 μm), bicuculline (10 μm), and strychnine (2 μm) at a holding potential of −40 mV in these 2 groups. The amplitude of NMDAR-EPSCs was also significantly increased in the presence of ZX1 (Fig 5G–5I). To investigate the potential involvement of presynaptic mechanisms in the effects of spinal Zn^2+^ on evoked excitatory postsynaptic currents (EPSCs), we employed paired-pulse ratio (PPR) analysis, a sensitive measure of presynaptic neurotransmitter release probability [46]. Chelation of spinal Zn^2+^ using ZX1 did not induce any significant changes in the PPR of EPSCs in NPY^+^ INs, indicating that presynaptic mechanisms do not contribute to the Zn^2+^-mediated attenuation of the EPSC amplitude (S7L–S7N Fig).

In the aged mice, both the amplitude of AMPAR-mediated and NMDAR-mediated EPSCs of NPY^+^ INs significantly reduced (S8A–S8D Fig). In dry skin model mice, the frequency of mEPSCs in NPY^+^ INs were dramatically reduced, while the amplitude did not change (S8E–S8I Fig). Chelation of spinal Zn^2+^ with ZX1 increased the amplitudes of both AMPAR-EPSCs and NMDAR-EPSCs (S8J–S8M Fig). These results are consistent with the notion that spinal Zn^2+^ inhibits both AMPAR- and NMDAR-mediated EPSCs and modulates the neuronal activity of NPY^+^ neurons via a postsynaptic mechanism. In sharp contrast, neither AMPAR-mediated EPSCs nor NMDAR-mediated EPSCs in Ucn3::Cre neurons were affected by chelation of endogenous Zn^2+^ with ZX1, indicating that Ucn3::Cre neurons was not directly inhibited by vesicular Zn^2+^ or that the inhibitory effect was not strong enough to affect the synaptic responses of Ucn3::Cre neurons (S9 Fig), which is also in line with the retrograde trans-monosynaptic tracing results (S6E and S6F Fig). Thus, these data provide evidence that Zn^2+^ released from presynaptic vesicles exerts a modulatory effect on the neuronal activity of NPY^+^ neurons through the inhibition of AMPARs and NMDARs.

## Discussion

Pruritus, the sensation of itching, is a prevalent skin complaint among individuals aged 65 and above [47,48]. In elderly individuals, particularly in autumn and winter, xerosis and dry skin are leading causes of itchiness characterized by heightened mechanical sensitivity [3,48]. In this study, we found that there is one subset of DRG neurons that express the vesicular Zn^2+^ transporter TMEM163 and are enriched with Zn^2+^. These neurons establish direct synaptic connections with NPY^+^ inhibitory INs in laminae III-IV of the dorsal horn. Notably, during aging, the expression of TMEM163 significantly increases, resulting in the accumulation of higher levels of Zn^2+^ within large-sized DRG neurons and the central terminals of large-sized DRG neurons within the spinal cord. Upon stimulation, excessive Zn^2+^ is released into synaptic clefts in the spinal dorsal horn, where it modulates the neuronal activity of NPY::Cre INs by inhibiting NMDARs and AMPARs, leading to the disinhibition of mechanical itch-associated neural circuitry and aging-related itch.

Under physiological conditions, mechanical itch is tightly regulated by spinal inhibitory INs, resulting in minimal perception [5,6,30]. TLR5^+^ Aβ-low-threshold mechanoreceptors (LTMRs)/Ucn3^+^ spinal neurons and NPY1R^+^ spinal neurons have been found to selectively transmit mechanical itch signals, which are strictly gated by neuropeptide Y (NPY)::Cre-derived inhibitory INs under physiological conditions. Mice with selective ablation of or silencing of spinal NPY^+^ INs experience more severe mechanical itch hypersensitivity and develop obvious scratch-induced skin lesions [6]. In contrast, chemogenetic activation of spinal NPY^+^ INs not only attenuates acute mechanical itch responses to light punctate stimulation of the skin behind the ears but also suppresses pruritogen-induced itch, reduces acute nocifensive reflexes, and alleviates behavioral symptoms of associated with neuropathic and inflammatory pain [3,7]. Gentle touch applied to the skin can simultaneously activate both the neural circuitry involved in transmitting mechanical itch (e.g., TLR5^+^ LTMRs to Ucn3^+^/Tac2^+^ INs) and an inhibitory neural circuitry that effectively suppresses mechanical itch. NPY^+^-derived INs have been identified as key elements involved in gating mechanical itch in the spinal cord [3,6]. However, the specific LTMRs that form monosynaptic connections with NPY^+^-derived INs remain unknown. Electrophysiological experiments conducted by Bourane and colleagues reveal that NPY^+^ INs in the spinal dorsal horn receive monosynaptic inputs from Aβ, Aδ, and C-fiber generating DRG neurons. The proportions of these monosynaptic inputs are 10.8% from Aβ, 40.5% from Aδ, and 48.7% from C-fiber DRG neurons, respectively [6]. However, our retrograde trans-monosynaptic tracing experiments revealed that NPY^+^ INs primarily receive monosynaptic inputs from DRG neuron expressing TMEM163, while they make few direct synaptic connections with Aδ-LTMRs and C-LTMRs. In contrast, only 27.74 ± 5.007% of the monosynaptic inputs to Ucn3^+^ interneurons were from TMEM163^+^ primary afferents. The findings by Albisetti and colleagues demonstrate a significant tropism for DRG neurons when rabies viruses are injected into the dorsal horn, with non-peptidergic nociceptors and C-LTMRs showing resistance to infection [49]. This can explain why our transsynaptic retrograde-traced neurons are mostly TMEM163^+^. Here, we can only state that NPY^+^-derived INs receive monosynaptic inputs from *Tmem163*^+^ primary afferents, whereas Ucn3::Cre neurons exhibit minimal monosynaptic connections with *Tmem163*^+^ primary afferents. Another point that should be clarified is that the cells revealed by the reporter cross are not necessarily the same as those captured by intraspinal injection of virus in adults. In the Science paper by Bourane and colleagues, they crossed the NPY::Cre transgenic mice with R26LSL-tdTomato; (Ai14) reporter mice to label INs that express NPY in the spinal dorsal horn. ISH with a probe against mouse *Npy* showed that only 35% of tdTomato^+^ neurons expressed NPY at P30 [6]. Thus, they concluded that NPY::Cre; Ai14 mice capture 2 populations of NPY-expressing neurons: one that transiently expresses NPY during late embryonic/early neonatal development and another that shows persistent expression into adulthood.

Zinc has been implicated in somatosensation, including pain, itch, and acid sensation, in several studies [36,50–52]. Zinc selectively activates TRPA1 via intracellular cysteine and histidine residues in its NH2-terminus [50]. TRPA1 is a nonselective cation channel located on the plasma membrane known to act as a sensor of pain, itch, and environmental irritants and is responsible for various protective responses such as tearing, airway resistance, and coughing [53,54]. Intradermal injection of Zn^2+^ at concentrations above 0.3 mM induces pronounced scratching behavior in mouse models of neck and cheek itch, and this effect is dependent on TRPA1 activation [36]. Another crucial nonselective cation channel involved in histamine-dependent chemical itch and various forms of chronic itch is TRPV1, which can be inhibited by Zn^2+^ [55]. However, the expression of TRPA1 and TRPV1 is limited to small peptidergic or nonpeptidergic DRG neurons with central branches terminating in laminae I-II of the spinal dorsal horn. Thus, it is less likely that zinc stored in large-sized DRG neurons and their central terminals regulates aging-related itch through TRPA1 and TRPV1. Additionally, vesicular Zn^2+^ stored in the central terminals of TMEM163^+^ primary afferents is released into the synaptic cleft upon neuronal depolarization. ZIP transporters, which are responsible for zinc influx from the extracellular space into the cytosol, are also expressed in large-sized DRG neurons, suggesting that Zn^2+^ may be recycled from the synaptic cleft back into the cytosol [36,41,51]. Therefore, it is less plausible that Zn^2+^ in the synaptic clefts in laminae III-V diffuses into the central terminals area of small DRG neurons expressing TRPV1 and TRPA1 in laminae I-II to regulate itch via modulating the channel activity of TRPV1 and TRPA1.

Both AMPARs and NMDARs can be inhibited by free Zn^2+^ [18,35]. Chelation of spinal Zn^2+^ with ZX1 or TPEN had no effect on mEPSCs but significantly increased the amplitudes of AMPAR- and NMDAR-mediated evoked EPSCs in NPY^+^ INs. Consistently, light touch-evoked itch decreased after chelation of spinal Zn^2+^ under naïve conditions. The data obtained from our study indicate that the spontaneous release of Zn^2+^ from presynaptic vesicles is inadequate to inhibit AMPARs and NMDARs on NPY^+^ INs. However, electrical stimuli and light touch are capable of triggering enhanced Zn^2+^ release into the synaptic cleft. Increased Zn^2+^ release may inhibit NPY^+^ INs, leading to a reduction in mechanical itch sensation. In contrast to NPY^+^ INs-ablated mice, which exhibit spontaneous itch and pronounced touch-evoked itch [3,6], young mice or mice in a naïve state exhibit only mild touch-evoked itch and low levels of spontaneous itch. This observation may be attributed to the relatively low concentration of Zn^2+^ present in the central terminals of large-sized DRG neurons, suggesting that the release of vesicular Zn^2+^ during spontaneous or even light touch stimulation is inadequate to fully inhibit the neuronal activity of NPY^+^ INs. However, in the presence of dry skin or during the aging process, TMEM163 expression in large-sized DRG neurons is notably up-regulated, and this change is accompanied by a substantial increase in the concentration of Zn^2+^ within the central terminals of these neurons. It is important to note that we did not verify whether the NPY promoter-driven mCherry expression was specific to NPY^+^ neurons when recorded activity in NPY neurons in both young and aged mice, due to the lack of a reliable NPY antibody in our laboratory. Nathanson and colleagues previously demonstrated that the NPY promoter, when driving GFP expression, is largely restricted to GABAergic neurons. However, other subtypes of inhibitory interneurons, such as PV, VIP, and SST neurons, can also express GFP to some extent [56]. Therefore, we cannot entirely rule out the possibility that our recordings included activity from other inhibitory neuron types in addition to NPY^+^ neurons. Given these considerations, we concluded that neuronal transmission to inhibitory interneurons is diminished in the spinal cord of aged mice. Furthermore, the application of light touch or engagement in grooming behavior can elicit release of Zn^2+^ into the synaptic cleft, resulting in the significant inhibition of NPY^+^-derived INs activity and subsequently leading to mechanical itch hypersensitivity. This may also explain the scratch-itch cycle observed during aging and in dry skin. However, the acute chemical itch induced by pruritogens such as CQ and 48/80 is unaffected by spinal Zn^2+^ chelation, suggesting that key spinal neurons, including those in the Nppb/GRP/GRPR system, may receive minimal primary sensory input from TMEM163^+^ afferents. Moreover, NPY^+^-derived interneurons appear to selectively gate the Ucn3 pathway but not the chemical itch system in the spinal cord. A study demonstrated that the loss of cutaneous touch receptors, specifically Merkel cells, leads to increased alloknesis during aging and in xerosis, indicating that inputs from SAI Aβ-LTMRs may attenuate the gating activity of NPY^+^ interneurons [6,30]. Furthermore, several studies have reported the involvement of NPY^+^-derived INs in pain gating [5]. Therefore, it is worth further investigating whether TMEM163^+^ afferents play a role in the development of mechanical pain hypersensitivity under inflammatory or neuropathic conditions, as the underlying mechanism remains unclear and is clinically important.

In summary, we identified a subpopulation of DRG neurons that express the vesicular Zn^2+^ transporter TMEM163. These neurons establish direct monosynaptic connections with NPY^+^-derived inhibitory INs. Notably, TMEM163 expression significantly increases with aging and in xerosis conditions, resulting in enhanced Zn^2+^ loading into presynaptic vesicles. Upon light touch stimulation, excessive release of Zn^2+^ into the synaptic cleft disrupts the inhibitory effect exerted by NPY^+^-derived inhibitory INs on mechanical itch-transmitting neurons, thereby facilitating the generation of both aging-related itch and xerosis-related itch (S10 Fig). The findings of this study provide novel mechanistic insight into the pathogenesis of these types of itch and highlight a promising therapeutic target for their management.

## Materials and methods

### Animals

*Tmem163*^*f/f*^ mice were procured from Cyagen Biosciences. The NPY::Cre mice (Stock No: 027851), VGluT1::Cre mice (Stock No: 023527), Ai3 mice (Stock No: 007903), and Ai14 mice (Stock No: 007908) were obtained from The Jackson Laboratory. Ucn3::Cre mice were generously provided by Dr. Haili Pan’s lab at Jiangxi Provincial People’s Hospital. C57BL/6 WT mice were purchased from Shanghai Lingchang Biological Technology Company. C57BL/6 aged mice were purchased from Jiangsu ALF Biotechnology Company.

All mice were group-housed and bred in the animal facilities of Fudan University, following a 12-h light/12-h dark cycle at a temperature of 22 ± 1°C. All behavioral tests were conducted by observers blind to the treatments and genotypes of animals. All mice used for behavior tests were genotyped and assigned to either experimental groups or control groups. All animal procedures were conducted in accordance with the guidelines outlined in the National Institutes of Health Guide for the Care and Use of Laboratory Animals and were approved by the Animal Care and Use Committee of the Institutes of Brain Science at Fudan University.

### ZnS^AMG^ staining

Mice intended for sampling were deeply anesthetized with 0.75% pentobarbital sodium at 10 μl/g body weight. After deep anesthesia, the mice were subjected to transcardiac perfusion with PBS followed by Na_2_S (FD Rapid TimmStain Kit, Cat: Pk701), followed by perfusion with 4% paraformaldehyde (PFA) supplemented with 0.1% picric acid in PBS as separate solutions. The DRG and spinal cord were subsequently excised, postfixed overnight at 4°C, and subjected to dehydration through a graded series of sucrose solutions. Serial transverse sections of the spinal cord (30 μm, free-floating) skin and DRG (14 μm) were prepared using a freezing microtome. The sections were thoroughly rinsed with 0.1 M phosphate buffer solution for 10 min and then immersed in an AMG incubation solution (containing 50% Arabic gelatin, sodium citrate buffer, p-diphenol, and silver lactate solution) for 35 min at 32°C. To ensure consistency of zinc reactions, tissue samples from different ages or groups were processed together in the same batch. For the dry skin model and dorsal root ligation experiments, treatments were applied to both sides of the same mice. This approach minimized variability caused by differences in processing times and conditions. All tissue samples were fixed, stained, and analyzed under identical conditions. Observers maintained blinding throughout the entire experimental and analysis processes to ensure the objectivity and consistency of data interpretation. Following dehydration and clearing procedures, the sections were sealed with neutral gum. Subsequently, brightfield microscopy images from ZnS^AMG^ staining section series were acquired using a Nikon microscope through a 20× objective. The white balance was adjusted prior to image capture. ZnS^AMG^ staining images were quantitatively analyzed using Image-Pro Plus 6.0 software. Before analysis, the optical density of the images was corrected, a white reference point was established, and the background was subtracted. Measurement regions were delineated using the irregular tool within the HSI color system. Subsequently, the total integrated optical density (IOD) was measured, and the mean density was calculated.

### Mice and human cerebrospinal fluid (CSF) collection and [Zn^2+^] test

Mice were deeply anesthetized with isoflurane, and the cisterna magna was surgically exposed. A sharpened glass electrode was affixed to a capillary holder, which was then connected to a 1-ml syringe using a plastic tube. Negative pressure was created in the glass electrode by pulling the syringe plunger back by 100~200 μl. Subsequently, the electrode was carefully inserted into the cisterna magna to collect CSF. The collected CSF samples were either stored in a −80°C freezer for future use or subjected to [Zn^2+^] tests using the Zinc Assay Kit (Sigma Aldrich Cat: MAK032) following the recommended procedure. After the CSF collection, mice were anesthetized with 0.75% pentobarbital sodium at 10 μl/g body weight and sacrificed by decapitation.

For human CSF collection, the study was approved by the Ethics Committee of Huashan Hospital of Fudan University with certificate No. KY2020-1218. The CSF collection was a voluntary CSF donation by the patients develop a single meningioma who consented to their CSF being used for research purpose in anonymized way. The written informed consent was received from participants prior to inclusion in the study. Eleven patients were divided into 2 groups based on their age: young group with age ≤38 years old and the old group with age ≥54 years old. The CSF samples were obtained through standard lumbar puncture and centrifuged at 4°C, and the supernatants were transferred to fresh tubes and stored at −80°C.

### In situ hybridization (ISH) and immunohistochemistry (IHC)

Mice were deeply anesthetized with isoflurane and subjected to transcardiac perfusion with PBS, followed by 4% PFA in 0.01 M PBS. The spinal cord was sectioned at a thickness of 30 μm, and the DRG was sectioned at a thickness of 14 μm using a cryostat (Leica). In situ hybridization (ISH) was performed using the RNAscope Fluorescent Multiplex Assay kit and probes specifically targeting mouse *Tmem163* (Cat No. 854531-C1), *Slc17a7*(Cat NO.416631-C2), and *Ntrk2* (Cat No.423611-C1) (Advanced Cell Diagnostics) in strict accordance with the recommended protocol. Subsequently, the sections were incubated with primary antibodies anti-SP (Guinea pig, 1:1,000; Neuromics; GP14103), anti-CGRP (goat, 1:1,000; Bio-Rad; AB_2290729), anti-VGluT1 (Guinea pig, 1:1,000; Millipore; AB_5905), anti-TH (rabbit, 1:1,000, Millipore; AB152), anti-NF200 (mouse, 1:1,000, Millipore; AB_477257), or Anti-NeuN (mouse, 1:1,000, Millipore; Cat No. MAB377) overnight at 4°C, followed by Cy3- (1:400, Jackson ImmunoResearch Laboratories Inc. Cat No.112-165-003), Cy5- (1:400, Jackson ImmunoResearch Laboratories Inc. Cat No.715-175-151), or FITC-conjugated secondary antibodies (1:400; Jackson ImmunoResearch Laboratories Inc. Cat No.115-095-003) or FITC-conjugated IB4 (10 μg/ml; Invitrogen. Cat No. I21411) or DAPI (1:1,000; Invitrogen; 62248), or Nissl (1:1,000; Invitrogen; N21483). Sections were mounted and examined under Olympus confocal laser scanning microscopy.

### Itch behavioral test

All mice were habituated to being placed in a testing box (8 × 8 × 20 cm^3) positioned on a metal mesh over 2 days in advance, with each habituation session lasting for 2 h. Subsequently, scratching behaviors were recorded using video surveillance for a duration of 1 h, in the absence of any observer. A scratch event was defined when a mouse lifted its hind paw to scratch the shaved region and subsequently returned the paw to either the floor or the mouth for licking. The recording of spontaneous itch behaviors was performed blindly and evaluated without knowledge of the experimental conditions.

For touch-evoked itch testing, the fur behind the ear or on the nape was shaved 2 days before the experiments. Mice were habituated for 30 min on 2 consecutive days in the behavioral testing apparatus. On the testing day, mice were placed in plastic enclosures and further habituated for 60 min. To further enhance data reproducibility and evaluate the effect of a single stimulation on subsequent scratch responses in mice, we administered 5 stimulations, with 5 s interval between each stimulation. We then recorded the total number of scratch bouts induced by these 5 stimulations as the score for touch-evoked itch in each mouse behind the ears and nape. Mechanical stimuli were applied using 0.07 g Von Frey filaments (North Coast Medical, Morgan Hill, California, United States of America). The total number of positive responses across the 5 stimuli was recorded to indicate the mechanical itch score. For chemical itch testing, mice were shaved on the nape of the neck and acclimated in the recording chambers for 2 consecutive days before behavioral testing. On the testing day, different chemicals were injected intradermally into the middle of the nape separately. Compound 48/80 (2 μg/μl, 50 μl, Sigma, C2313), chloroquine (4 μg/μl, 50 μl, Sigma, C6628), β-alanine (60 μg/μl, 50 μl, Macklin, B874460), and SLGRL (2 μg/μl, 50 μl, tgpeptide) in a volume of 50 μl saline were injected intradermally. The number of scratching events was counted for 30 min after the injection. All behavioral tests were done by observers blind to the treatments or genotypes of animals.

### Dry skin model

Fur on the nape was removed 2 days before treatment. To generate a dry skin model, the nape was subjected to a treatment involving a mixture of acetone/ether (1:1, Sigma, St. Louis, Missouri, USA) for 20 s, followed by distilled water for 40 s (referred to as AEW), twice daily, for 7 consecutive days. The spontaneous itch was recorded using a camera for 1 h following the protocol for itch behavioral testing. Mechanical itch hypersensitivity was assessed using a 0.07 g Von Frey filament, as previously described.

### Dorsal root ligation model

Mice were anesthetized with pentobarbital sodium (75 mg/kg i.p.). The hair on the back, ranging from lumbar (L) 3 to lumbar (L) 6, was removed using a hair remover. Mice were then fixed in a stereotaxic instrument (RWD Lift Science Co.), L4-L5 region was shaved and wiped with iodophor, and the skin and muscle were cut and removed with a scissor to expose the vertebral plate, zygapophysis, and transverse process of spine column. Using a cranial drill (RWD Lift Science Co.) carefully removed the left part of the L4 and L5 vertebral plate, zygapophysis, and transverse process to expose the L4 and L5 DRG. Tying the dorsal roots of L4-L5 with fine thread, disinfecting and suturing the skin, and collecting samples 3 days later.

### Intrathecal injection of drugs

ZX1 (Cat. No: 07–0350) was purchased from Strem chemicals, TPEN (Cat. No: P4413), ZnCl_2_ (Cat. No: 746355) were ordered from Sigma. TPEN were dissolved in 5% dimethyl sulfoxide (DMSO). Unless otherwise specified, other reagents are dissolved in sterile saline. Intrathecal injection of drugs or siRNA was performed as described previously (Wang and colleagues). For *i*.*t*. injection TPEN (100 μm) and ZnCl_2_ (100 μm), a spinal cord puncture was made with a 30-gauge needle between L5 and L6 levels to deliver drugs (10 μl) to the CSF, and then performed behavioral tests after 1 h.

### qRT-PCR

The mice were anesthetized and decapitated; DRG tissue was harvested and ground into powder using zirconia beads. Total RNAs were extracted using Trizol reagent. Reverse transcription of RNAs (0.5 to 2 μg) was performed using the 5 × All in One RT MasterMix (abm, G492). Power SYBR Green PCR Master Mix (Thermo 4367659) was used for the qRT-PCR experiment. For testing the expression of *Tmem163* in the dorsal root ganglia (DRG) of wild-type (WT) mice and *Tmem163* cKO mice, utilizing the following primer sequences: Forward primer: 5′-actgtctccgtcatgagg-3′, Reverse primer: 5′-agagtgcacagcagccgc-3′. The Real-time PCR was conducted following the protocol for Power SYBR Green Master Mix kit with primers presented in S1 Table. The expression levels of ZnTs were normalized to GAPDH using the 2^-ΔCt^ method.

### Genotyping

To purify genomic DNA, mouse ear tissue was collected and digested in a solution containing 25 mM NaOH and 0.2 mM EDTA at 95°C for 1 h, followed by neutralization with 40 mM Tris-HCl (pH 5.5). Genotyping of Tmem163 flox mice was performed using the primer pair 5′-agggcccttatatatctcactgtaa-3′ and 5′-aaaaccacctgatgttgaactgtg-3′, resulting in a WT band size of 132 bp and a mutant band size of 202 bp. The primers for genotyping NPY::cre mice were 5′-acaccggccttattccaag-3′, 5′-tccatgatttgcctcttgtg-3′, and 5′-ctgagacatagattcgtccaagg-3′ with band size 100 bp. The primers for genotyping VGluT1::cre mice were 5′-ccctaggaatgctcgtcaag-3′, 5′-atgagcgag gagaagtgtgg-3′, and 5′-gtggaagtcctggaaactgc-3′ with band size 344 bp.

### Plasmid construction and AAV construction

The full-length sequence of VGluT1 was amplified from mouse cDNA and cloned into pcDNA3.1, resulting in the generation of QE15-pcDNA3.1-VGluT1-mCherry. Truncations of VGluT1 were created using the KOD-Plus-Mutagenesis Kit (TOYOBO) and In-Fusion HD cloning kit (Takara). The pAKD-CMV-bGlobin-Tmem163-eGFP plasmid was obtained from Genechem company. Plasmids for Glycerol bacterium were provided by Genechem company for AAV construction. The coding sequence of Tmem163 was amplified from mouse cDNA and cloned into CMV-bGlobin-MCS-eGFP-3flag-Wpre-hGH-polyA. The synthesis and packaging of the AAV9-CMV-bGlobin-mTMEM163-EGFP-3FLAG-WPRE-hGH poly A virus were performed by Genechem company. AAV infection was achieved by intrathecal injection into the spinal cord.

### Cell culture and plasmid transfection

Primary cultures of DRG neurons were prepared following the established protocol described previously. DRGs were dissected from postnatal day 0 to 10 (P0-P10) mice and subjected to digestion using collagenase (1 mg/ml, Sigma-Aldrich), trypsin (0.4 mg/ml, Sigma-Aldrich), and deoxyribonuclease I (0.1 mg/ml) for 45 min. Subsequently, non-neuronal cells were removed by centrifugation with 15% Percoll, and the purified DRG neurons were obtained. The neurons were then cultured in neurobasal medium supplemented with 2% B27 supplement, 2 mM L-glutamine (Invitrogen), and 100 ng/ml nerve growth factor (NGF; Invitrogen). The cells were seeded onto poly-D-lysine-coated glass cover slips and maintained in a neurobasal defined medium at 37°C with 5% CO_2_ for 48 h before conducting experiments. The ND7/23 cell line was obtained from the Cell Bank of the Chinese Academy of Sciences. ND7/23 cells were cultured in DMEM media (without L-glutamine) supplemented with 10% FBS, 1% L-glutamine, and 1% PS at 37°C under 5% CO_2_. Plasmid transfection was performed using Lipofectamine 3000 (Invitrogen) according to the manufacturer’s instructions. For electroporation of DRG neurons, cells were suspended in electroporation buffer and then electroporated using the Nucleofector II (Amaxa) with the O-003 program.

### Live-cell imaging and data analysis

P10 DRG neurons that underwent electroporation were cultured on glass-bottom dishes placed on a Nikon temperature-controlled workstation maintained at 37°C. Live imaging of the neurons was performed using NIS-Elements viewer software. Specifically, axon segments located 100 to 150 μm from the cell body were carefully selected as regions of interest, and a total of 360 frames were acquired over a 6-min period. Subsequently, videos and individual frames were generated using Imaries software. The analysis of puncta was conducted using Image-Pro Plus 6.0 software.

### Immunoelectron microscopy

Recombinant adenovirus AAV9-*Tmem163*-EGFP was generated and packaged for subsequent intrathecal injection. Following the injection, mice were perfused with a solution containing 4% PFA and 0.2% glutaraldehyde to facilitate post-embedding immunogold labeling. Vibratome sections from the cervical spinal cord segments were then post-fixed in 0.5% osmium tetroxide and processed for embedding in resin. Ultrathin sections of the dorsal horn were prepared and subjected to staining with specific antibodies. Specifically, mouse anti-GFP antibodies (1:200, Earthox, E22030-03) and guinea pig anti-VGluT1 antibodies (1:200, Millipore, Ab5905) were used. Immunogold particles measuring 10 nm (anti-mouse, 1:40; Amersham) and 6 nm (anti-guinea pig, 1:40; Jackson) were applied. The selection of different diameters of colloidal gold particles depended on the content of the molecules in the tissue and their activity after embedding. Subsequently, uranium staining was performed to visualize the localization of *Tmem163* and VGluT1 molecules expressed on the vesicles under an electron microscope. To exclude nonspecific signals, we conducted 2 control experiments: a negative control and a no primary antibody control. For the negative control, naïve C57 mice were sampled and subjected to the same immunoelectron microscopy procedure described above. Ultrathin sections of the dorsal horn were stained with mouse anti-GFP and guinea pig anti-VGluT1 antibodies. Immunogold particles measuring 10 nm and 6 nm were used to label GFP and VGluT1, respectively. For the no primary antibody control, AAV9-*Tmem163*-EGFP infected mice were sampled and subjected to the same immunoelectron microscopy procedure described above. Ultrathin sections of the dorsal horn were incubated with primary antibody dilution buffer, followed by 10 nm and 6 nm immunogold particles.

### Intra-spinal injection

Mice were anesthetized with pentobarbital sodium (75 mg/kg i.p.), and their eyes were covered with erythromycin eye ointment to prevent ophthalmic drying. The hair on the back, ranging from thoracic (T) 10 to lumbar (L) 2, was removed using a hair remover. Mice were then fixed in a stereotaxic instrument (RWD Lift Science Co.), and the spinal cord between T11-T12, T12-T13, or T13-L1 was exposed. A glass micropipette connected to Nanoject III (was purchased from Drummond Scientific Company) was utilized for injection. The injection site was tilted at a 30-degree angle and positioned 500 μm away from the midline, which could be identified by the blood vessel running along the longitudinal axis of the spinal cord. The surface of the spinal cord served as the starting point for the Z-axis, and the micropipette was pulled back 200 μm, resulting in the settlement of the micropipette tip 300 μm from the dorsal surface. A total volume of 300 nl of viral particles was injected at a rate of 60 nl/min. Following the injection, the micropipette was held in place for 5 min to prevent virus leakage. To identify the presynaptic neurons that directly project to NPY^+^ INs, a viral mixture consisting of 300 nl, containing equal volumes of AAV2/5-hEF1a-DIO-RVG-WPRE-pA (Taitool, S0325-5, 2.20E+12 vg/ml) and AAV2/9-CAG-DIO-TVA-mCherry-WPRE-pA (Taitool, S0683-9, 4.50E+11 vg/ml) was injected. The incision was closed using 4–0 sutures. Three weeks later, 300 nl of RV-ENVA-DG-EGFP (BrainVTA, R01002, 2.00E+08 IFU/ml) was injected at the same site. After 1 week, mice were sacrificed for histochemical analysis. To label spinal NPY^+^ interneurons in aged mice or young control mice, rAAV2/9-NPY-mCherry-WPRE-bGH polyA (BrainVTA, PT1216, titer: 6.05E+12 vg/ml) was delivered via intraspinal injection into the spinal L2-L4 regions of both young and aged C57/BL6 mice. After 4 weeks, the mice were sacrificed for whole-cell patch clamp recordings.

### Spinal cord slice preparation and patch-clamp recordings

Mice were deeply anesthetized with pentobarbital sodium (75 mg/kg, i.p.). Transcardiac perfusion of ice-cold modified artificial cerebrospinal fluid (ACSF) containing (in mM): KCl 2, NaH_2_PO_4_ 1.3, CaCl_2_ 0.2, MgSO_4_ 12, NaHCO_3_ 26, D-glucose 10, sucrose 220, sodium ascorbate 1.3, sodium pyruvate 3.5 (oxygenated with 95% O_2_ and 5% CO_2_, pH 7.4). The spinal cord slices from lumbar segments 4 to 5 were prepared in the transverse plane by quickly dissecting the lumbar spinal cord, placing it into ice-cold modified ACSF, and cutting it at 300 μm using a vibratome (VT1000S, Leica, Germany). Slices were incubated in a solution containing (in mM): NaCl 126, KCl 2.5, CaCl_2_ 2, MgCl_2_ 1, NaH_2_PO_4_ 1.25, NaHCO_3_ 26, D-glucose 10, sodium ascorbate 1.3, sodium pyruvate 3.5 (oxygenated with 95% O_2_ and 5% CO_2_) for 30 min and at RT for 1 h. The slices were transferred into a recording chamber and perfused with oxygenated recording solution at a rate of 5 ml/min prior to electrophysiological recordings at RT.

Whole-cell patch clamp recordings were performed on laminae III-V NPY^+^ neurons in voltage-clamp mode. Patch pipettes (5 to 10 M) from borosilicate glass (Sutter, USA) were made on a horizontal micropipette puller (P-1000, Sutter, USA). Data were acquired with pClamp10.0 software (Molecular Devices, USA) using MultiClamp700B patch-clamp amplifier and Digidata1550B (Molecular Devices, USA). For minimal excitatory postsynaptic current (mEPSC) recording, pipette solution contained (in mM): Cs-methanesulfonate 120, CsCl 15, Na2 creatine pgosphate 10, HEPES 10, Mg-ATP 2, Na-GTP 0.3, QX-314 2.5, EGTA 1, with pH at 7.3 and measured osmolarity at 300 to 310 mOsm. After establishing the whole-cell configuration, neurons were holding at −70 mV to record mEPSCs in the presence of bicuculline (100 μm), strychnine (2 μm), and TTX (0.5 μm). AMPA receptor-mediated EPSCs were elicited by electrical stimulation in the presence of bicuculline (10 mM) and strychnine (2 mM). NMDA receptor-mediated EPSCs were recorded using a low Mg^2+^ recording ACSF (2.5 mM Ca^2+^, 0.25 mM Mg^2+^) supplemented with NBQX (10 μm), bicuculline (10 μm), and strychnine (2 μm). The holding potential was set at −40 mV, as specified. EPSCs were evoked by applying a constant current pulse at 0.05 Hz. The signals were filtered at 3 kHz and digitized at 10 kHz.

### Ethics statement

All experiments were approved by the Experimental Animal Ethics Committee of Shanghai Medical College and the Committee on the Use of Animal Experiments of Fudan University (approval number: SYXK2020-0032). For human CSF collection, the study was approved by the Ethics Committee of Huashan Hospital, Fudan University (approval number: KY2020-1218). The samples were voluntarily donated by patients diagnosed with solitary meningioma, who provided written informed consent for the anonymous use of their cerebrospinal fluid in research. The study was conducted in compliance with the principles outlined in the Declaration of Helsinki.

### Quantification and statistical analysis

All data are presented as bar graphs indicating mean ± SEM (standard error of the mean). Dots in bar graphs and boxplots represent individual values per mouse or per image, horizontal line indicates average. GraphPad Prism scientific software version 8.4.0 was used for statistical analysis. For normally distributed, related samples with equal variances, unpaired *t* test and paired *t* test was used to compare 2 groups. Conversely, samples with unequal variances via Welch’s *t* test. Statistical significance was determined using both nonparametric (Mann–Whitney, Kruskal–Wallis test with Dunn’s multiple-comparisons post hoc test) and parametric methods (one-way ANOVA followed by Dunnett’s multiple comparisons, two-way ANOVA with Sidak’s multiple comparisons Bonferroni post hoc analysis) due to differences in the normality and sample size of the data. The specific tests used to analyze each data set is indicated within the individual figure legends, with results expressed as mean ± SEM. Significance levels are indicated as *p < 0.05, **p < 0.01, ***p < 0.001.

## Supporting information

S1 Fig Increased Zn^2+^ levels in the skin under aged conditions. **(A, B)** ZnS^AMG^ staining in the nape skin of young mice and aged mice. (A) Representative image of ZnS^AMG^ staining in nape skin. Scale bar = 50 μm. (B) Quantitative analysis of the mean intensity of ZnS^AMG^ staining of the young and aged mice; unpaired *t* test, *n* = 20–21 slices/group. All data are expressed as mean ± SEM. ****p < 0.0001. The underlying data for S1B Fig can be found in S1 Data.
(TIFF)

S2 Fig Aged mice develop both spontaneous itch and touch-evoked itch. (**A**) Schematic diagram showing the measurement of itch responses to 0.07 g Von Frey filament applied to the back of the ear and nape in mice. (**B**) Spontaneous itch responses of WT mice of different ages. One-way ANOVA followed by Dunnett’s multiple comparisons. *n* = 8 mice/group; *n* = 5–9 mice/group. (**C**) Itch responses to 0.07 g Von Frey filament applied to the back of the ear in WT mice of different ages. Unpaired *t* test; *n* = 5–10 mice/group. (**D**) Itch responses to 0.07 g Von Frey filament applied to the nape in WT mice of different ages. Kruskal–Wallis test with Dunn’s multiple-comparisons test; *n* = 6–10 mice/group. **(E)** Spontaneous itch response after intrathecal injection of vehicle or the Zn^2+^ chelator ZX1 in 18-month-old mice. Unpaired *t* test; *n* = 6–7 mice/group. **(F)** Touch-evoked itch response after intrathecal injection of vehicle or the Zn^2+^ chelator ZX1 in 18-month-old mice. Unpaired *t* test; *n* = 6–7 mice/group. **(G, H)** ZnS^AMG^ staining in the skin tissues (nape) after intrathecal injection of vehicle or the Zn^2+^ chelator TPEN in 2-month-old mice. (G) Representative image of ZnS^AMG^ staining in skin (nape). Scale bar = 50 μm. (H) Quantitative analysis of the mean intensity of ZnS^AMG^ staining of the Vehicle and TPEN groups; unpaired *t* test, *n* = 20–19 slices/group. **(I, J)** ZnS^AMG^ staining in the skin tissues (nape) after intrathecal injection of vehicle or ZnCl_2_ in 2-month-old mice. (I) Representative image of ZnS^AMG^ staining in skin (nape). Scale bar = 50 μm. (J) Quantitative analysis of the mean intensity of ZnS^AMG^ staining of the Vehicle and ZnCl_2_ groups; unpaired *t* test, *n* = 20–19 slices/group. All data are expressed as mean ± SEM. *p < 0.05, **p < 0.01, ***p < 0.001. The underlying data for S2B, S2C, S2D, S2E, S2F, S2H, and S2J Fig can be found in S1 Data.
(TIFF)

S3 Fig The expression of ZnTs in the DRG and the role of TMEM163 (ZnT11) in mechanical itch. (**A**) Relative expression of ZnT1 (*Slc30a1*)-ZnT10 (*Slc30a10*) and TMEM163 in different subpopulations of DRG neurons based on a published single-cell database (GSE59739). (**B**) ISH using a probe targeting mouse *Tmem163* combined with IHC staining for FITC-IB4 (nonpeptidergic DRG neurons), CGRP (peptidergic DRG neurons), SP (peptidergic DRG neurons), and TH (C-fiber low-threshold mechanoreceptors). Scale bar = 100 μm. (**C**) Double FISH using probes targeting mouse *Tmem163* (red) and mouse *Slc17a7* (green). Scale bar = 100 μm. (**D**) Itch responses of *Tmem163*^*f/f*^ mice and *Tmem163* cKO mice in response to touch stimulation by different filaments; two-way ANOVA followed by Sidak’s multiple comparisons test; *n* = 6 mice/group. **(E**) Touch-evoked itch responses of *Tmem163*^*f/f*^ mice and *Tmem163* cKO mice following AEW-mediated induction of dry skin; unpaired *t* test; *n* = 6 mice/group. All data are expressed as the mean ± SEM; ***p < 0.001. The underlying data for S3D and S3E Fig can be found in S1 Data.
(TIFF)

S4 Fig The colocalization between VGluT1 and TMEM163 in presynaptic vesicles. **(A)** Negative control, ultrathin sections from naïve C57 mice were stained with antibodies (mouse anti-GFP; guinea pig anti-VGluT1) and immunogold particles measuring 10 nm and 6 nm. The arrow indicates a VGluT1-positive vesicle. Scale bar = 200 nm for left panel and scale bar = 100 nm for right panel. **(B)** No primary antibody control, ultrathin sections from AAV9-Tmem163-EGFP infected mice incubated with primary antibody dilution buffer, followed by incubation with 10 nm immunogold particles. Scale bar = 200 nm for left panel and scale bar = 100 nm for right panel. **(C)** Quantification of colocalized 6 nm and 10 nm colloidal gold particles in presynaptic vesicles. Data are expressed as the mean ± SEM; *n* = 27 slices/group. The underlying data for S4C Fig can be found in S1 Data.
(TIFF)

S5 Fig Distribution of NPY::Cre IN neurons within the spinal cord. (**A**) Double staining of spinal cord slices from NPY::Cre;Ai3 mice with primary antibodies against CGRP and DAPI staining of these slices. Representative images showing the colocalization of CGRP (red) with DAPI (blue). Scale bars = 200 μm (left) and 20 μm (right). (**B**) Double staining of VGluT1 (red) and DAPI (blue) in spinal dorsal horn slices from NPY::Cre;Ai3 mice. Scale bar = 50 μm.
(TIFF)

S6 Fig Cell type characterization of neurons upstream of NPY::Cre INs in the DRG. (**A**) Representative image of a spinal dorsal horn slice from an NPY::Cre mouse infected with the retrograde transsynaptic tracing virus. Scale bar = 10 μm. (**B**) Representative images showing the colocalization of EGFP with *Ntrk2* (ISH), NF200 (IHC staining), and TH (IHC staining) in presynaptic neurons. Scale bar = 100 μm. (**C**) The percentage of NF200^+^, *Ntrk2*^*+*^ and TH^+^ DRG neurons that were presynaptic NPY::Cre IN (EGFP^+^). The data are expressed as the mean ± SEM; *n* = 9–10/group. (**D**) Staining of spinal cord slices from UCN3::Cre;Ai3 mice with an anti-CGRP primary antibody and FITC-IB4 antibody and DAPI staining of these slices. Scale bar = 100 μm. (**E**) Representative images showing the infected presynaptic neurons (EGFP^+^) of Ucn3::Cre INs in DRG and coexpression with *Tmem163* by using ISH. Scale bar = 100 μm. (**F**) Quantitative analysis of the data in (E). All data are expressed as the mean ± SEM. The underlying data for S6C and S6F Fig can be found in S1 Data.
(TIFF)

S7 Fig Effect of vesicular Zn^2+^ on mEPSCs in NPY::Cre INs. (**A**) Schematic diagram of the procedure used to record mEPSCs in NPY::Cre INs. (**B**) Representative mEPSCs in NPY::Cre INs in laminae III-V before and after TPEN application. (**C**) The frequency of mEPSCs in NPY::Cre INs in laminae III-V before and after TPEN application; paired *t* test; *n* = 8 cells. (**D**) The cumulative probability of different mEPSC frequencies in NPY::Cre INs in laminae III-V before and after TPEN application; *n* = 8 cells. (**E**) The amplitude of mEPSCs in NPY::Cre INs in laminae III-V before and after TPEN application; paired *t* test, *n* = 8 cells. (**F**) The cumulative probability of different mEPSC amplitudes of NPY::Cre INs in laminae III-V before and after TPEN application; *n* = 8 cells. (**G**) Representative mEPSCs in NPY::Cre INs in laminae III-V before and after ZX1 application. (**H**) The frequency of mEPSCs in NPY::Cre INs in laminae III-V before and after ZX1 application; paired *t* test; *n* = 11 cells. (**I**) The cumulative probability of different mEPSC frequencies in NPY::Cre INs in laminae III-V before and after ZX1 application; *n* = 11 cells. (**J**) The amplitude of mEPSCs in NPY::Cre INs in laminae III-V before and after ZX1 application; paired *t* test; *n* = 11 cells. (**K**) The cumulative probability of different mEPSC amplitudes in NPY::Cre INs in laminae III-V before and after ZX1 application; *n* = 11 cells. (**L**) Representative eEPSCs in laminae III-V NPY::Cre INs in response to 2 stimuli at 50 ms intervals before and after ZX1 application. (**M**) Time course of the change in the paired-pulse ratio before and after ZX1 application; *n* = 9 cells. (**N**) Quantitative analysis of the paired-pulse ratio; paired *t* test; *n* = 9 cells/group. All data are expressed as the mean ± SEM; ns: not statistically significant. The underlying data for S7C, S7D, S7E, S7F, S7H, S7I, S7J, S7K, S7M, and S7N Fig can be found in S1 Data.
(TIFF)

S8 Fig The neuronal activity of NPY::Cre INs under dry skin and aged conditions. **(A, B)** Example traces (A) and summarized data (B) of AMPAR-EPSC on spinal cord laminar IIi-V NPY^+^ neurons of young or aged mice. **(C, D)** Example traces (C) and summarized data (D) of NMDAR-EPSC on spinal cord laminar IIi-V NPY^+^ neurons of young or aged mice. (**E**) Representative mEPSCs in NPY::Cre INs in laminae III-V in naïve mice and dry skin model mice. (**F**) The frequency of mEPSCs in NPY::Cre INs in laminae III-V in naïve mice and dry skin model mice; unpaired *t* test; *n* = 17–19 cells. (**G**) The cumulative probability of different mEPSC frequencies in NPY::Cre INs in laminae III-V, *n* = 17–19 cells. (**H**) The amplitude of mEPSCs in NPY::Cre INs in laminae III-V; Mann–Whitney test; *n* = 17–19 cells. (**I**) The cumulative probability of different mEPSC amplitudes in NPY::Cre INs in laminae III-V; *n* = 17–19 cells. (**J**) Representative eEPSCs in laminae III-V NPY::Cre INs of dry skin model mice before and after ZX1 application (hold at −70 mV). (**K**) Quantitative analysis of the eEPSC amplitude; paired *t* test; *n* = 10 cells/group. (**L**) Representative eEPSCs in laminae III-V NPY::Cre INs of dry skin model mice before and after ZX1 application (hold at −40 mV). (**M**) Quantitative analysis of the eEPSC amplitude; paired *t* test; *n* = 8 cells/group. All data are expressed as the mean ± SEM; *p < 0.05, **p < 0.01, ***p < 0.001. The underlying data for S8B, S8D, S8F, S8G, S8H, S8I, S8K, and S8M Fig can be found in S1 Data.
(TIFF)

S9 Fig Effect of vesicular Zn^2+^ on eEPSCs in Ucn3::Cre INs. (**A**) Schematic diagram of the procedure used to record eEPSCs in Ucn3::Cre INs. (**B**) Representative eEPSCs in Ucn3::Cre INs in laminae II-III before and after ZX1 application (hold at −70 mV). (**C**) Time course of the change in eEPSC amplitude before and after ZX1 application; *n* = 6 cells. (**D**) Quantitative analysis of the eEPSC amplitude; paired *t* test; *n* = 6 cells. (**E**) Representative eEPSCs in Ucn3::Cre INs in laminae II-III before and after ZX1 application (hold at −40 mV). (**F**) Time course of the change in eEPSC amplitude before and after ZX1 application; *n* = 7 cells. (**G**) Quantitative analysis of the eEPSC amplitude; paired *t* test; *n* = 7 cells. All data are expressed as the mean ± SEM; n.s.: not statistically significant. The underlying data for S9C, S9D, S9F, and S9G Fig can be found in S1 Data.
(TIFF)

S10 Fig Working model for TMEM163^+^ primary afferents mediating age-associated itch. The central branch of TMEM163^+^ primary afferents projects to the deep laminae of the spinal dorsal horn and selectively synapses onto NPY^+^ INs. In young mice or under normal condition, the expression level of TMEM163 in large-sized DRG neurons is low, resulting in the minimal accumulation of vesicular Zn^2+^ in the central terminal of TMEM163^+^ primary afferents. Consequently, light touch elicits a low release of Zn^2+^ and less inhibition of NPY^+^ INs. However, in aged mice or under dry skin condition, the expression of TMEM163^+^ significantly increases, leading to elevated accumulation of Zn^2+^ in the central terminal of TMEM163^*+*^ primary afferents. This heightened expression results in more Zn^2+^ released into the synaptic cleft during light stimulation, causing increased inhibition of NPY^+^ INs through AMPARs and NMDARs.
(TIFF)

S1 Table Primer list. (DOCX)

S1 Raw Images Supplemental raw image. (TIF)

S1 Data Supplemental raw data. (XLSX)

## Abbreviations

AAV: adeno-associated virus
ACSF: artificial cerebrospinal fluid
AEW: acetone–ether–water
cKO: conditional knockout
CSF: cerebrospinal fluid
DCN: dorsal cochlear nucleus
DMSO: dimethyl sulfoxide
DRG: dorsal root ganglia
EPSC: excitatory postsynaptic current
GRPR: gastrin-releasing peptide receptor
IHC: immunohistochemistry
IN: interneuron
IOD: integrated optical density
ISH: in situ hybridization
LTMR: low-threshold mechanoreceptor
mEPSC: minimal excitatory postsynaptic current
NF: neurofilament
NGF: nerve growth factor
PFA: paraformaldehyde
PPR: paired-pulse ratio
SEM: standard error of the mean
WT: wild-type
TH: tyrosine hydroxylase
