# Supplementary figures and images for: Elevated vesicular Zn2+ in dorsal root ganglion neurons expressing the transporter TMEM163 causes age-associated itchy skin in mice
Source: PLoS Biol. 2024 Nov 27;22(11):e3002888. doi: 10.1371/journal.pbio.3002888 (PMC11602076; doi:10.1371/journal.pbio.3002888)

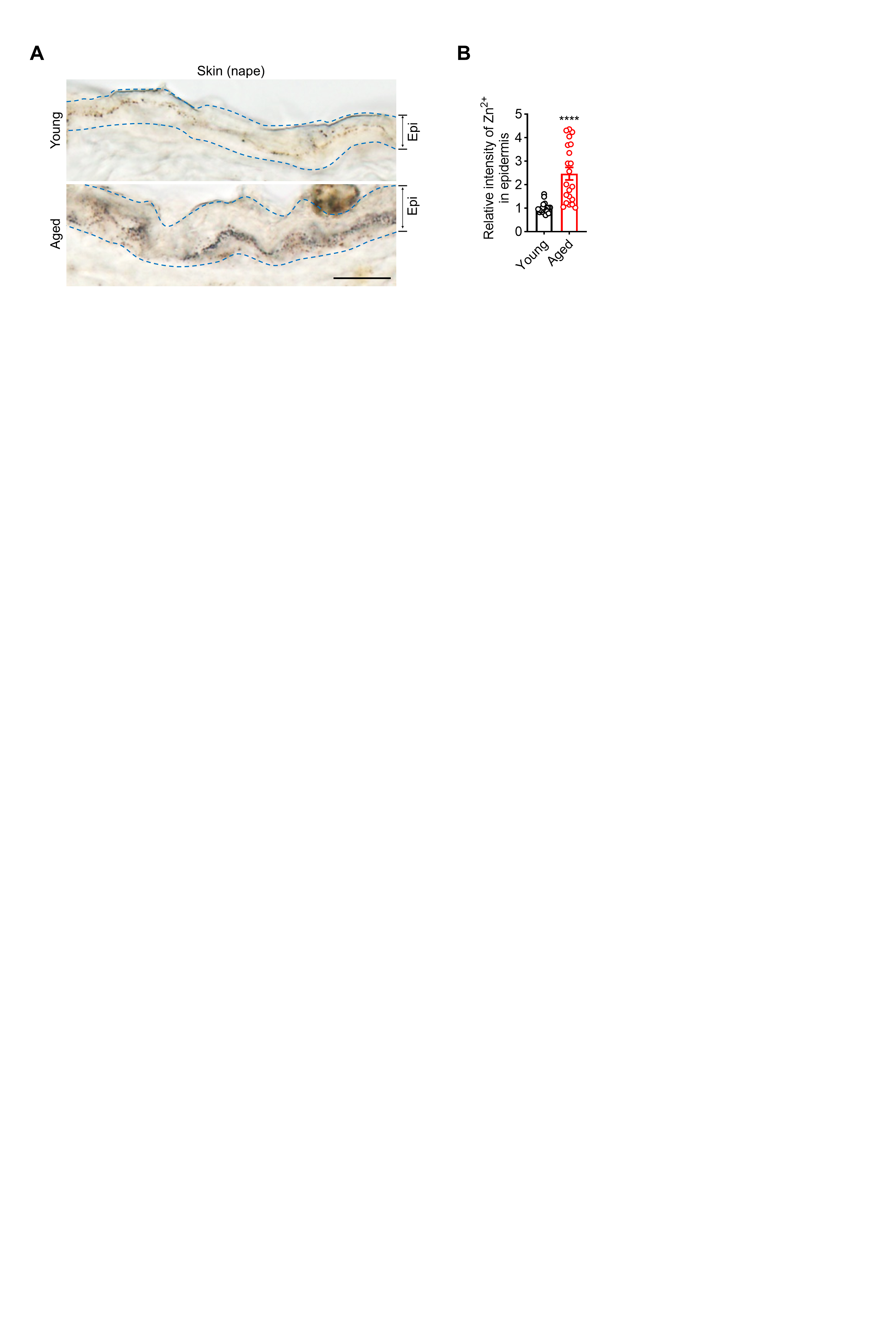

Supplement: S1 Fig — (A, B) ZnSAMG staining in the nape skin of young mice and aged mice. (A) Representative image of ZnSAMG staining in nape skin. Scale bar = 50 μm. (B) Quantitative analysis of the mean intensity of ZnSAMG staining of the young and aged mice; unpaired t test, n = 20–21 slices/group. All data are expressed as mean ± SEM. ****p < 0.0001. The underlying data for S1B Fig can be found in S1 Data. (TIFF) [file pbio.3002888.s001.tiff]

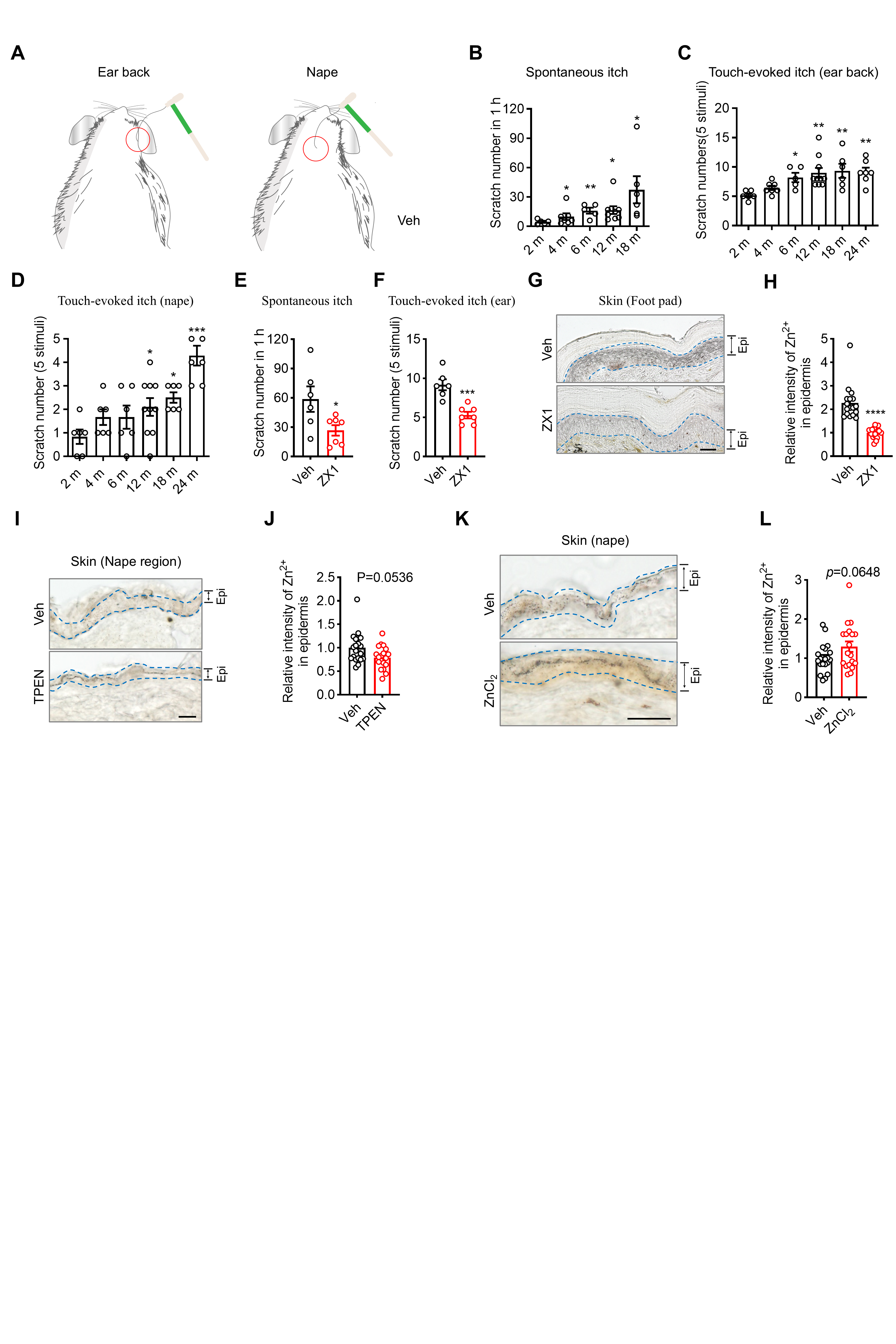

Supplement: S2 Fig — (A) Schematic diagram showing the measurement of itch responses to 0.07 g Von Frey filament applied to the back of the ear and nape in mice. (B) Spontaneous itch responses of WT mice of different ages. One-way ANOVA followed by Dunnett’s multiple comparisons. n = 8 mice/group; n = 5–9 mice/group. (C) Itch responses to 0.07 g Von Frey filament applied to the back of the ear in WT mice of different ages. Unpaired t test; n = 5–10 mice/group. (D) Itch responses to 0.07 g Von Frey filament applied to the nape in WT mice of different ages. Kruskal–Wallis test with Dunn’s multiple-comparisons test; n = 6–10 mice/group. (E) Spontaneous itch response after intrathecal injection of vehicle or the Zn2+ chelator ZX1 in 18-month-old mice. Unpaired t test; n = 6–7 mice/group. (F) Touch-evoked itch response after intrathecal injection of vehicle or the Zn2+ chelator ZX1 in 18-month-old mice. Unpaired t test; n = 6–7 mice/group. (G, H) ZnSAMG staining in the skin tissues (nape) after intrathecal injection of vehicle or the Zn2+ chelator TPEN in 2-month-old mice. (G) Representative image of ZnSAMG staining in skin (nape). Scale bar = 50 μm. (H) Quantitative analysis of the mean intensity of ZnSAMG staining of the Vehicle and TPEN groups; unpaired t test, n = 20–19 slices/group. (I, J) ZnSAMG staining in the skin tissues (nape) after intrathecal injection of vehicle or ZnCl2 in 2-month-old mice. (I) Representative image of ZnSAMG staining in skin (nape). Scale bar = 50 μm. (J) Quantitative analysis of the mean intensity of ZnSAMG staining of the Vehicle and ZnCl2 groups; unpaired t test, n = 20–19 slices/group. All data are expressed as mean ± SEM. *p < 0.05, **p < 0.01, ***p < 0.001. The underlying data for S2B, S2C, S2D, S2E, S2F, S2H, and S2J Fig can be found in S1 Data. (TIFF) [file pbio.3002888.s002.tiff]

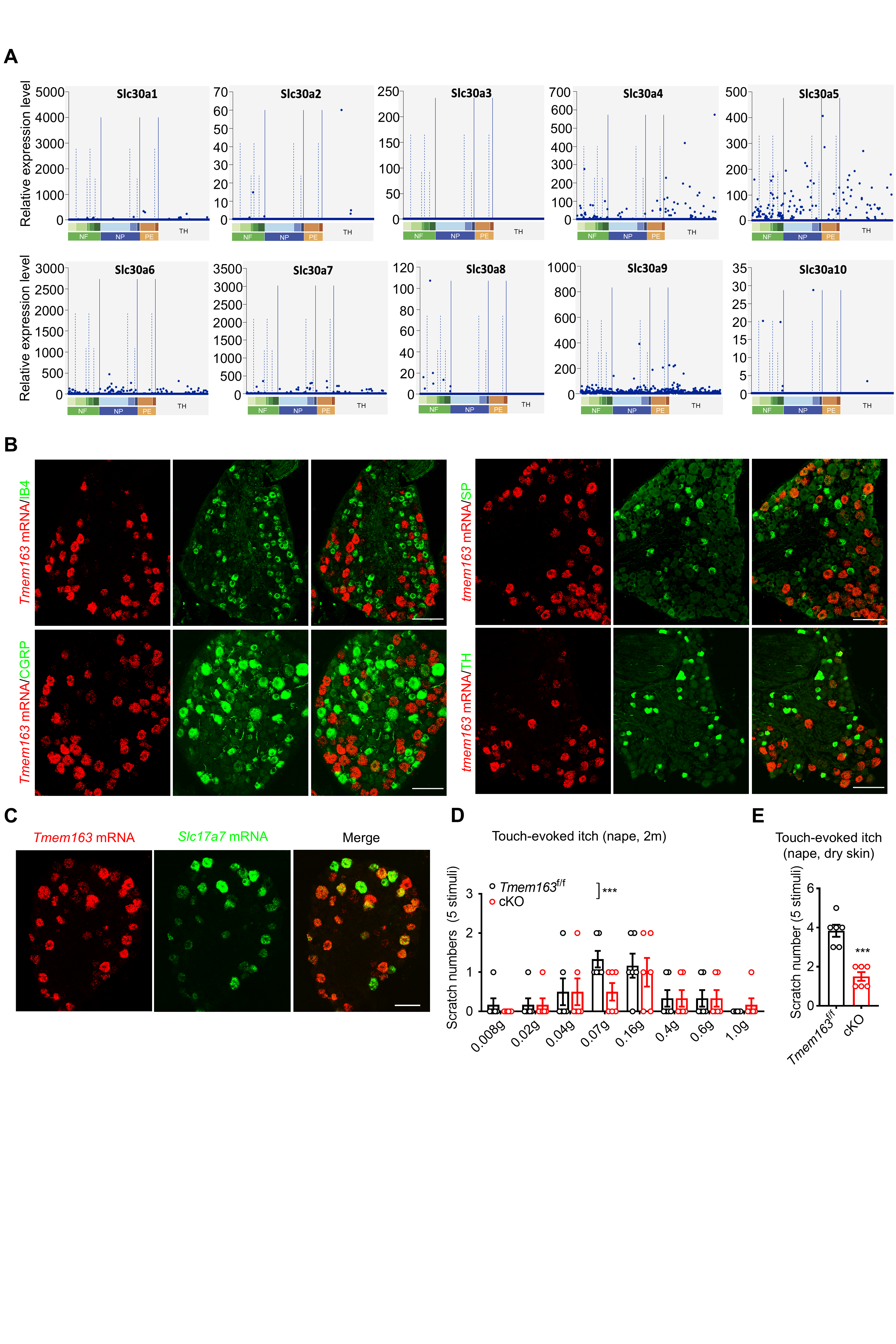

Supplement: S3 Fig — (A) Relative expression of ZnT1 (Slc30a1)-ZnT10 (Slc30a10) and TMEM163 in different subpopulations of DRG neurons based on a published single-cell database (GSE59739). (B) ISH using a probe targeting mouse Tmem163 combined with IHC staining for FITC-IB4 (nonpeptidergic DRG neurons), CGRP (peptidergic DRG neurons), SP (peptidergic DRG neurons), and TH (C-fiber low-threshold mechanoreceptors). Scale bar = 100 μm. (C) Double FISH using probes targeting mouse Tmem163 (red) and mouse Slc17a7 (green). Scale bar = 100 μm. (D) Itch responses of Tmem163f/f mice and Tmem163 cKO mice in response to touch stimulation by different filaments; two-way ANOVA followed by Sidak’s multiple comparisons test; n = 6 mice/group. (E) Touch-evoked itch responses of Tmem163f/f mice and Tmem163 cKO mice following AEW-mediated induction of dry skin; unpaired t test; n = 6 mice/group. All data are expressed as the mean ± SEM; ***p < 0.001. The underlying data for S3D and S3E Fig can be found in S1 Data. (TIFF) [file pbio.3002888.s003.tiff]

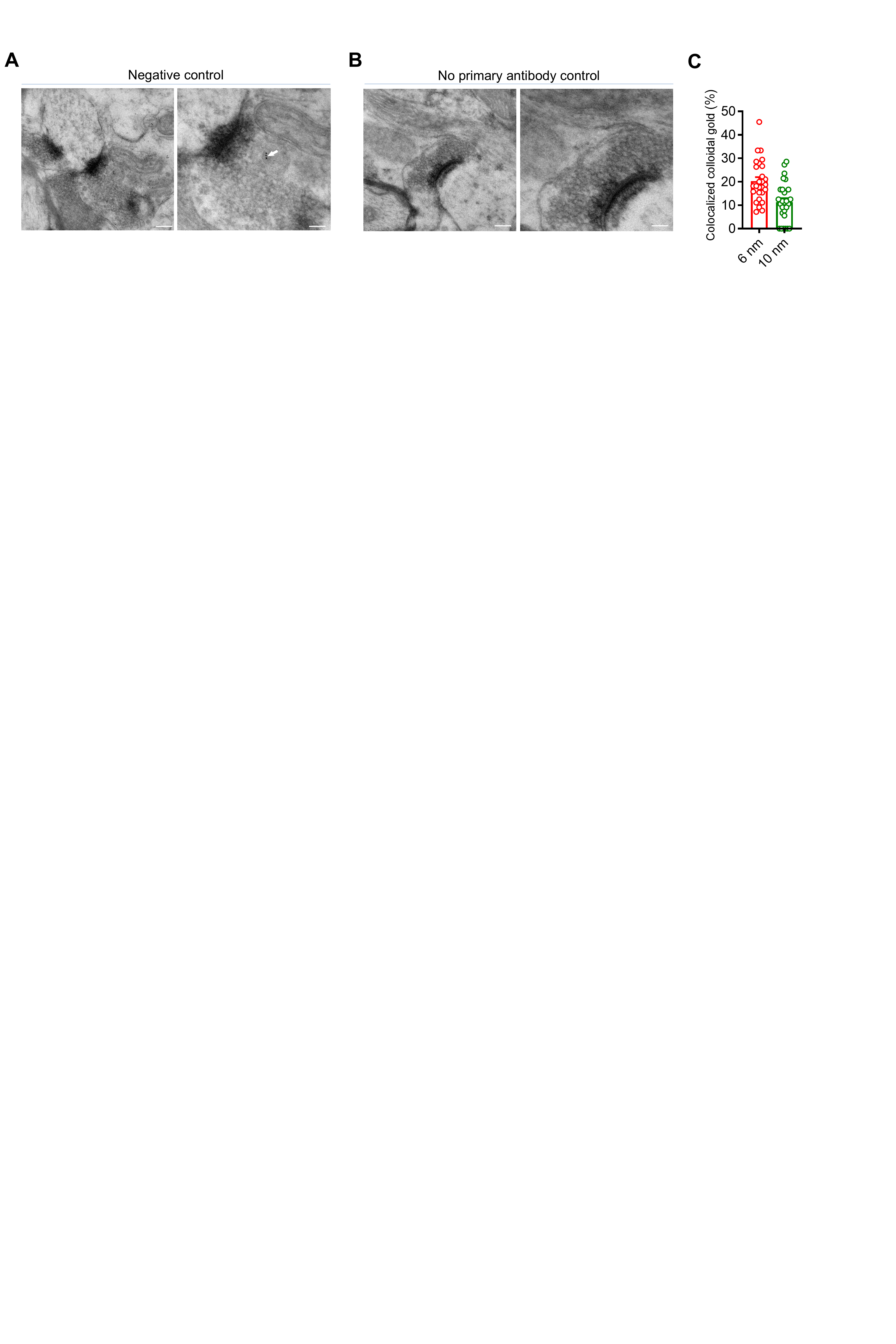

Supplement: S4 Fig — (A) Negative control, ultrathin sections from naïve C57 mice were stained with antibodies (mouse anti-GFP; guinea pig anti-VGluT1) and immunogold particles measuring 10 nm and 6 nm. The arrow indicates a VGluT1-positive vesicle. Scale bar = 200 nm for left panel and scale bar = 100 nm for right panel. (B) No primary antibody control, ultrathin sections from AAV9-Tmem163-EGFP infected mice incubated with primary antibody dilution buffer, followed by incubation with 10 nm immunogold particles. Scale bar = 200 nm for left panel and scale bar = 100 nm for right panel. (C) Quantification of colocalized 6 nm and 10 nm colloidal gold particles in presynaptic vesicles. Data are expressed as the mean ± SEM; n = 27 slices/group. The underlying data for S4C Fig can be found in S1 Data. (TIFF) [file pbio.3002888.s004.tiff]

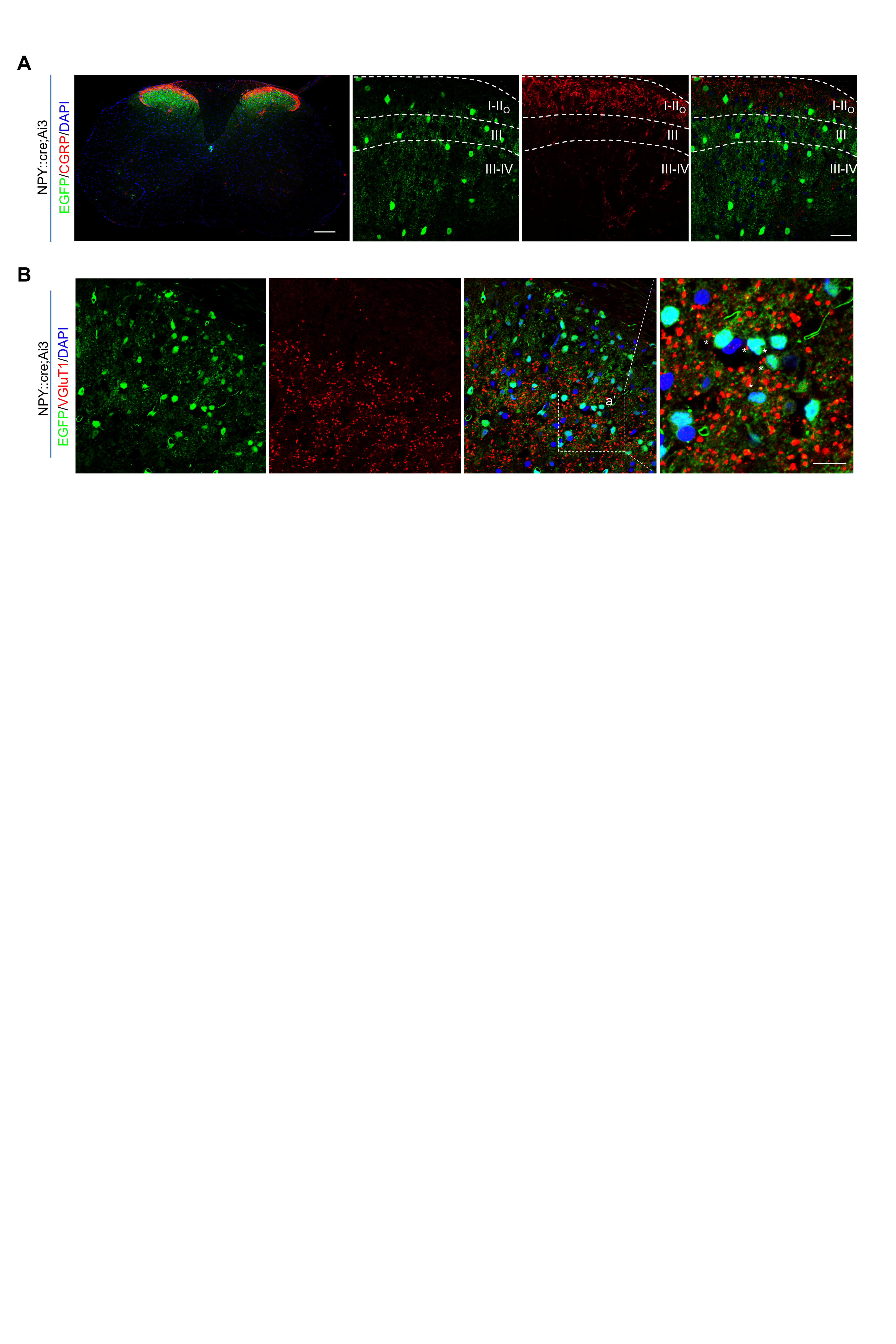

Supplement: S5 Fig — (A) Double staining of spinal cord slices from NPY::Cre;Ai3 mice with primary antibodies against CGRP and DAPI staining of these slices. Representative images showing the colocalization of CGRP (red) with DAPI (blue). Scale bars = 200 μm (left) and 20 μm (right). (B) Double staining of VGluT1 (red) and DAPI (blue) in spinal dorsal horn slices from NPY::Cre;Ai3 mice. Scale bar = 50 μm. (TIFF) [file pbio.3002888.s005.tiff]

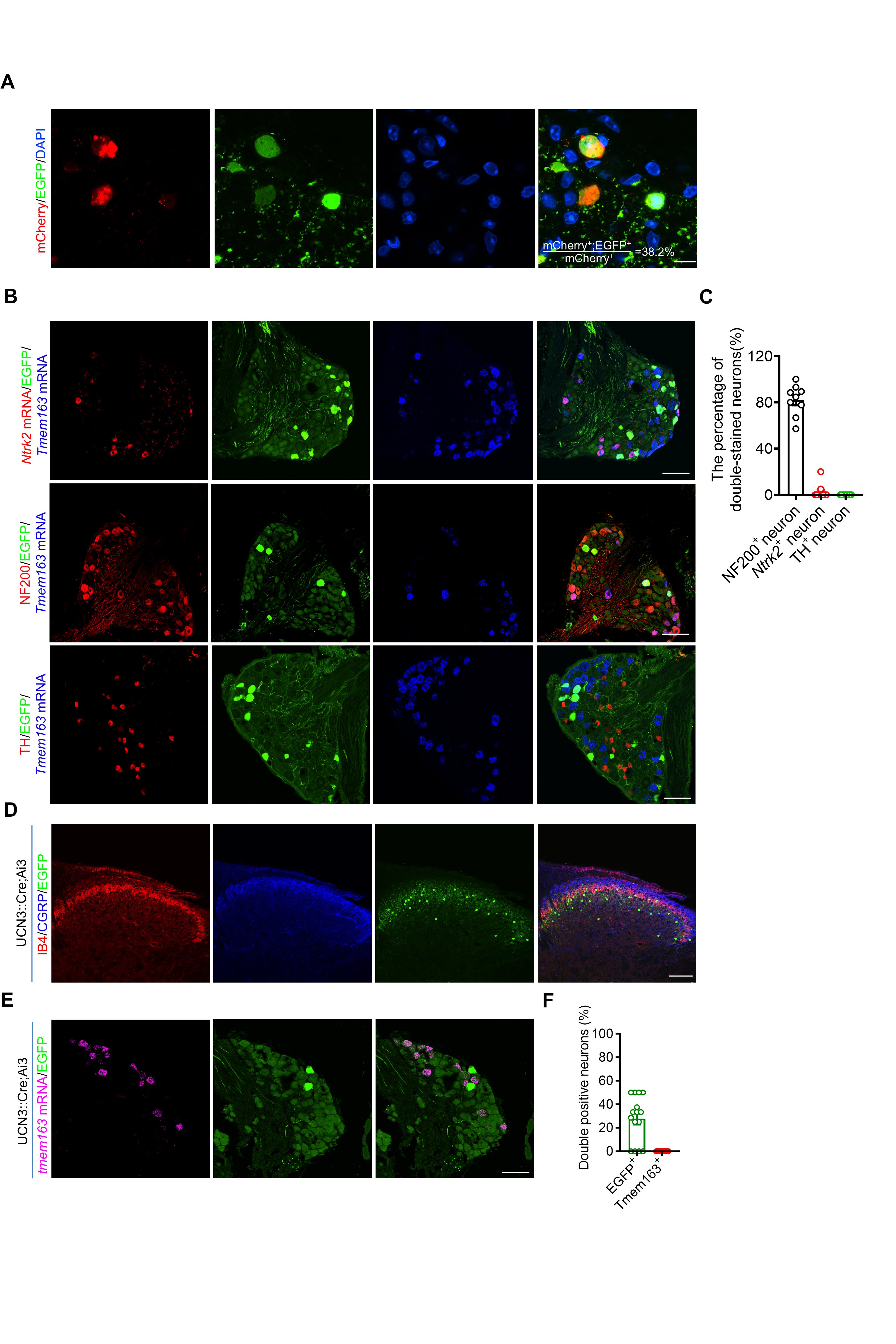

Supplement: S6 Fig — (A) Representative image of a spinal dorsal horn slice from an NPY::Cre mouse infected with the retrograde transsynaptic tracing virus. Scale bar = 10 μm. (B) Representative images showing the colocalization of EGFP with Ntrk2 (ISH), NF200 (IHC staining), and TH (IHC staining) in presynaptic neurons. Scale bar = 100 μm. (C) The percentage of NF200+, Ntrk2+ and TH+ DRG neurons that were presynaptic NPY::Cre IN (EGFP+). The data are expressed as the mean ± SEM; n = 9–10/group. (D) Staining of spinal cord slices from UCN3::Cre;Ai3 mice with an anti-CGRP primary antibody and FITC-IB4 antibody and DAPI staining of these slices. Scale bar = 100 μm. (E) Representative images showing the infected presynaptic neurons (EGFP+) of Ucn3::Cre INs in DRG and coexpression with Tmem163 by using ISH. Scale bar = 100 μm. (F) Quantitative analysis of the data in (E). All data are expressed as the mean ± SEM. The underlying data for S6C and S6F Fig can be found in S1 Data. (TIFF) [file pbio.3002888.s006.tiff]

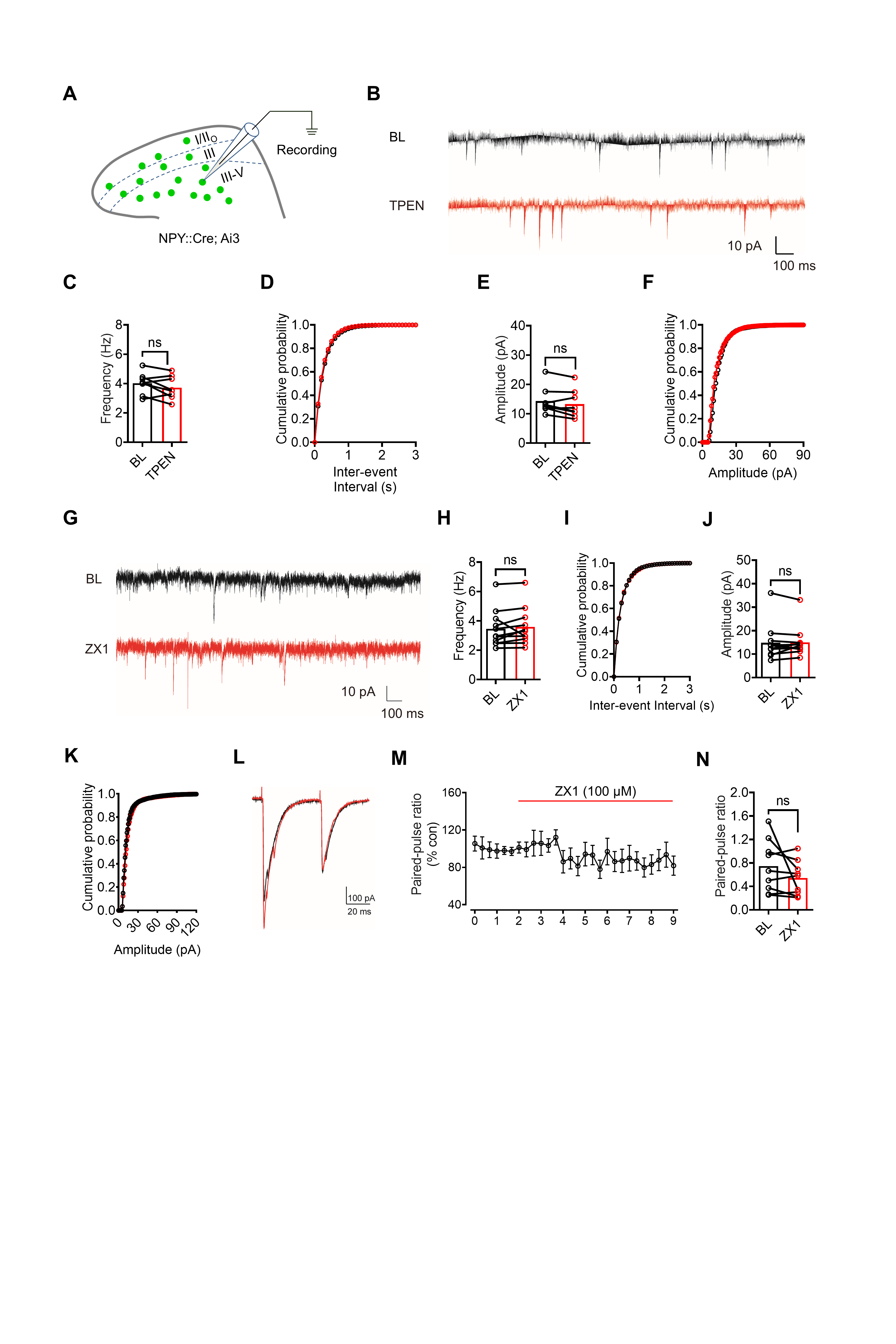

Supplement: S7 Fig — (A) Schematic diagram of the procedure used to record mEPSCs in NPY::Cre INs. (B) Representative mEPSCs in NPY::Cre INs in laminae III-V before and after TPEN application. (C) The frequency of mEPSCs in NPY::Cre INs in laminae III-V before and after TPEN application; paired t test; n = 8 cells. (D) The cumulative probability of different mEPSC frequencies in NPY::Cre INs in laminae III-V before and after TPEN application; n = 8 cells. (E) The amplitude of mEPSCs in NPY::Cre INs in laminae III-V before and after TPEN application; paired t test, n = 8 cells. (F) The cumulative probability of different mEPSC amplitudes of NPY::Cre INs in laminae III-V before and after TPEN application; n = 8 cells. (G) Representative mEPSCs in NPY::Cre INs in laminae III-V before and after ZX1 application. (H) The frequency of mEPSCs in NPY::Cre INs in laminae III-V before and after ZX1 application; paired t test; n = 11 cells. (I) The cumulative probability of different mEPSC frequencies in NPY::Cre INs in laminae III-V before and after ZX1 application; n = 11 cells. (J) The amplitude of mEPSCs in NPY::Cre INs in laminae III-V before and after ZX1 application; paired t test; n = 11 cells. (K) The cumulative probability of different mEPSC amplitudes in NPY::Cre INs in laminae III-V before and after ZX1 application; n = 11 cells. (L) Representative eEPSCs in laminae III-V NPY::Cre INs in response to 2 stimuli at 50 ms intervals before and after ZX1 application. (M) Time course of the change in the paired-pulse ratio before and after ZX1 application; n = 9 cells. (N) Quantitative analysis of the paired-pulse ratio; paired t test; n = 9 cells/group. All data are expressed as the mean ± SEM; ns: not statistically significant. The underlying data for S7C, S7D, S7E, S7F, S7H, S7I, S7J, S7K, S7M, and S7N Fig can be found in S1 Data. (TIFF) [file pbio.3002888.s007.tiff]

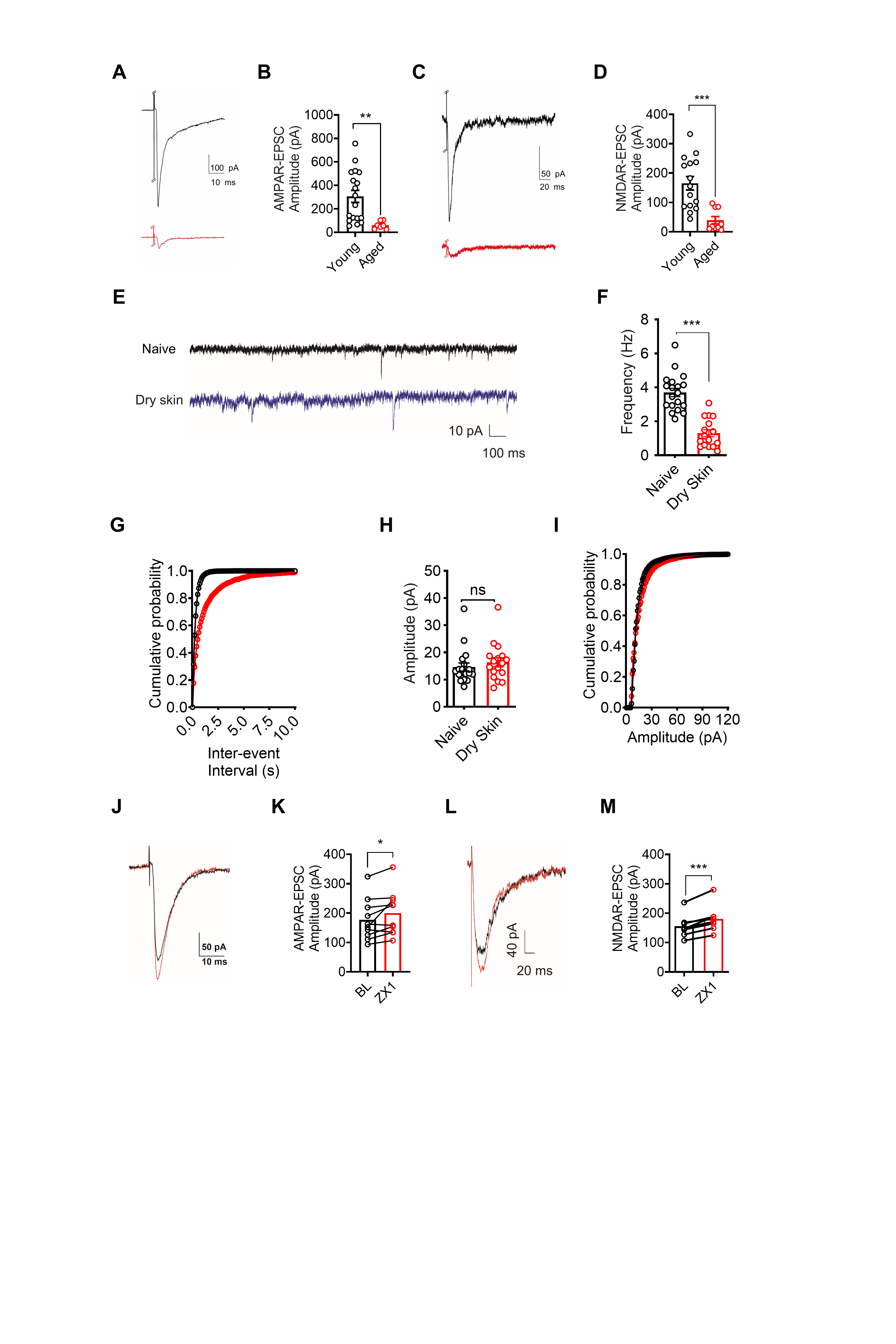

Supplement: S8 Fig — (A, B) Example traces (A) and summarized data (B) of AMPAR-EPSC on spinal cord laminar IIi-V NPY+ neurons of young or aged mice. (C, D) Example traces (C) and summarized data (D) of NMDAR-EPSC on spinal cord laminar IIi-V NPY+ neurons of young or aged mice. (E) Representative mEPSCs in NPY::Cre INs in laminae III-V in naïve mice and dry skin model mice. (F) The frequency of mEPSCs in NPY::Cre INs in laminae III-V in naïve mice and dry skin model mice; unpaired t test; n = 17–19 cells. (G) The cumulative probability of different mEPSC frequencies in NPY::Cre INs in laminae III-V, n = 17–19 cells. (H) The amplitude of mEPSCs in NPY::Cre INs in laminae III-V; Mann–Whitney test; n = 17–19 cells. (I) The cumulative probability of different mEPSC amplitudes in NPY::Cre INs in laminae III-V; n = 17–19 cells. (J) Representative eEPSCs in laminae III-V NPY::Cre INs of dry skin model mice before and after ZX1 application (hold at −70 mV). (K) Quantitative analysis of the eEPSC amplitude; paired t test; n = 10 cells/group. (L) Representative eEPSCs in laminae III-V NPY::Cre INs of dry skin model mice before and after ZX1 application (hold at −40 mV). (M) Quantitative analysis of the eEPSC amplitude; paired t test; n = 8 cells/group. All data are expressed as the mean ± SEM; *p < 0.05, **p < 0.01, ***p < 0.001. The underlying data for S8B, S8D, S8F, S8G, S8H, S8I, S8K, and S8M Fig can be found in S1 Data. (TIFF) [file pbio.3002888.s008.tiff]

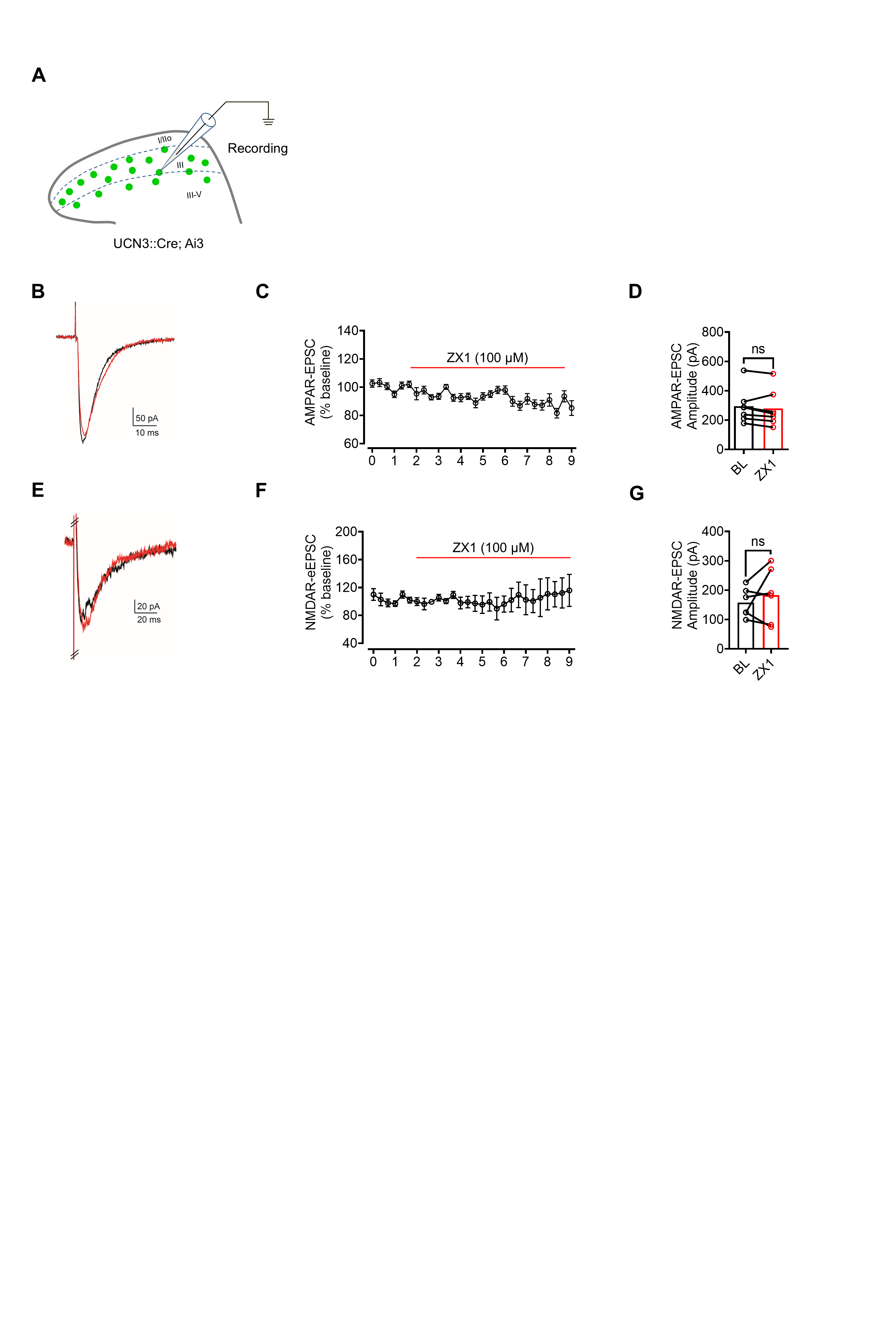

Supplement: S9 Fig — (A) Schematic diagram of the procedure used to record eEPSCs in Ucn3::Cre INs. (B) Representative eEPSCs in Ucn3::Cre INs in laminae II-III before and after ZX1 application (hold at −70 mV). (C) Time course of the change in eEPSC amplitude before and after ZX1 application; n = 6 cells. (D) Quantitative analysis of the eEPSC amplitude; paired t test; n = 6 cells. (E) Representative eEPSCs in Ucn3::Cre INs in laminae II-III before and after ZX1 application (hold at −40 mV). (F) Time course of the change in eEPSC amplitude before and after ZX1 application; n = 7 cells. (G) Quantitative analysis of the eEPSC amplitude; paired t test; n = 7 cells. All data are expressed as the mean ± SEM; n.s.: not statistically significant. The underlying data for S9C, S9D, S9F, and S9G Fig can be found in S1 Data. (TIFF) [file pbio.3002888.s009.tiff]

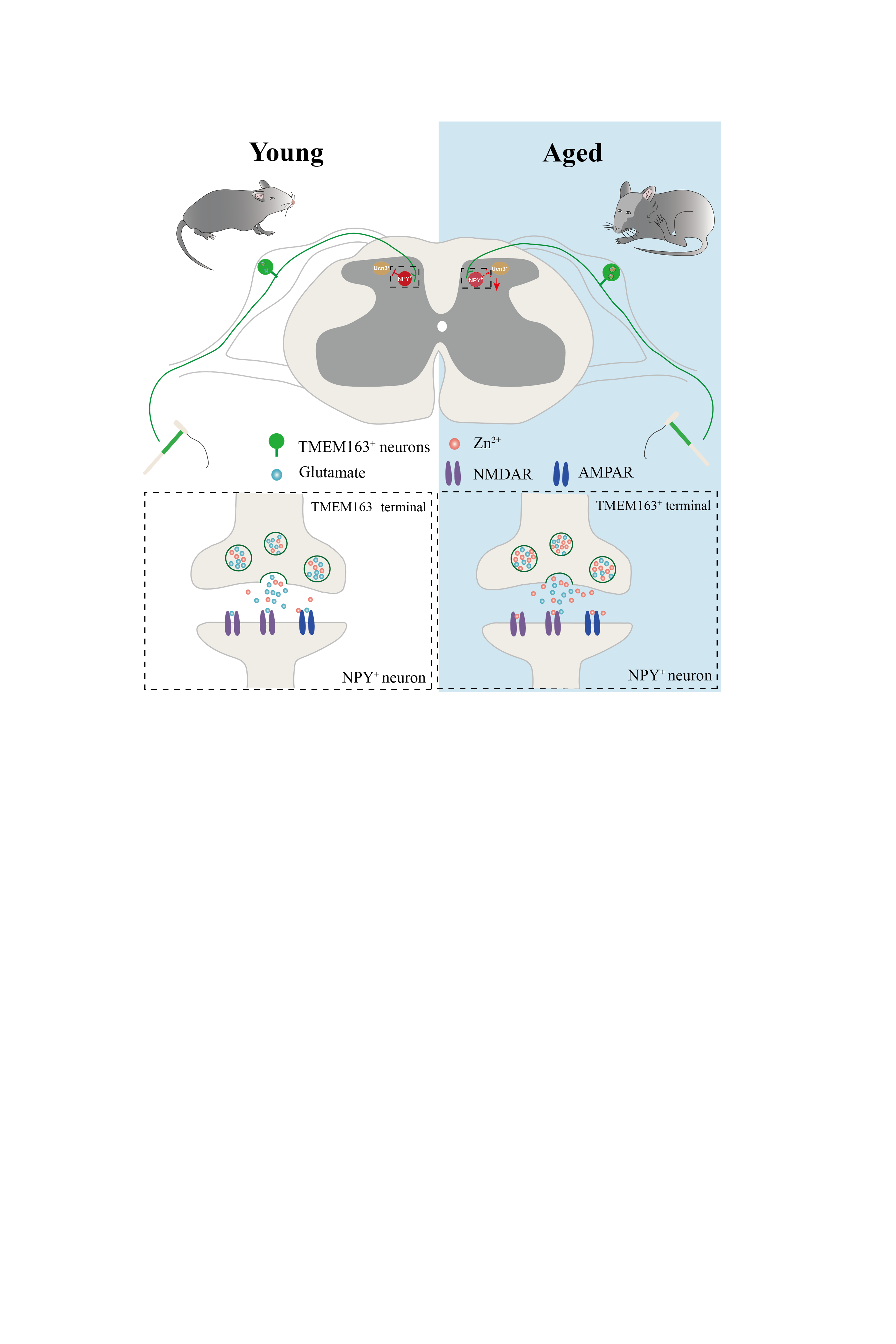

Supplement: S10 Fig — The central branch of TMEM163+ primary afferents projects to the deep laminae of the spinal dorsal horn and selectively synapses onto NPY+ INs. In young mice or under normal condition, the expression level of TMEM163 in large-sized DRG neurons is low, resulting in the minimal accumulation of vesicular Zn2+ in the central terminal of TMEM163+ primary afferents. Consequently, light touch elicits a low release of Zn2+ and less inhibition of NPY+ INs. However, in aged mice or under dry skin condition, the expression of TMEM163+ significantly increases, leading to elevated accumulation of Zn2+ in the central terminal of TMEM163+ primary afferents. This heightened expression results in more Zn2+ released into the synaptic cleft during light stimulation, causing increased inhibition of NPY+ INs through AMPARs and NMDARs. (TIFF) [file pbio.3002888.s010.tiff]

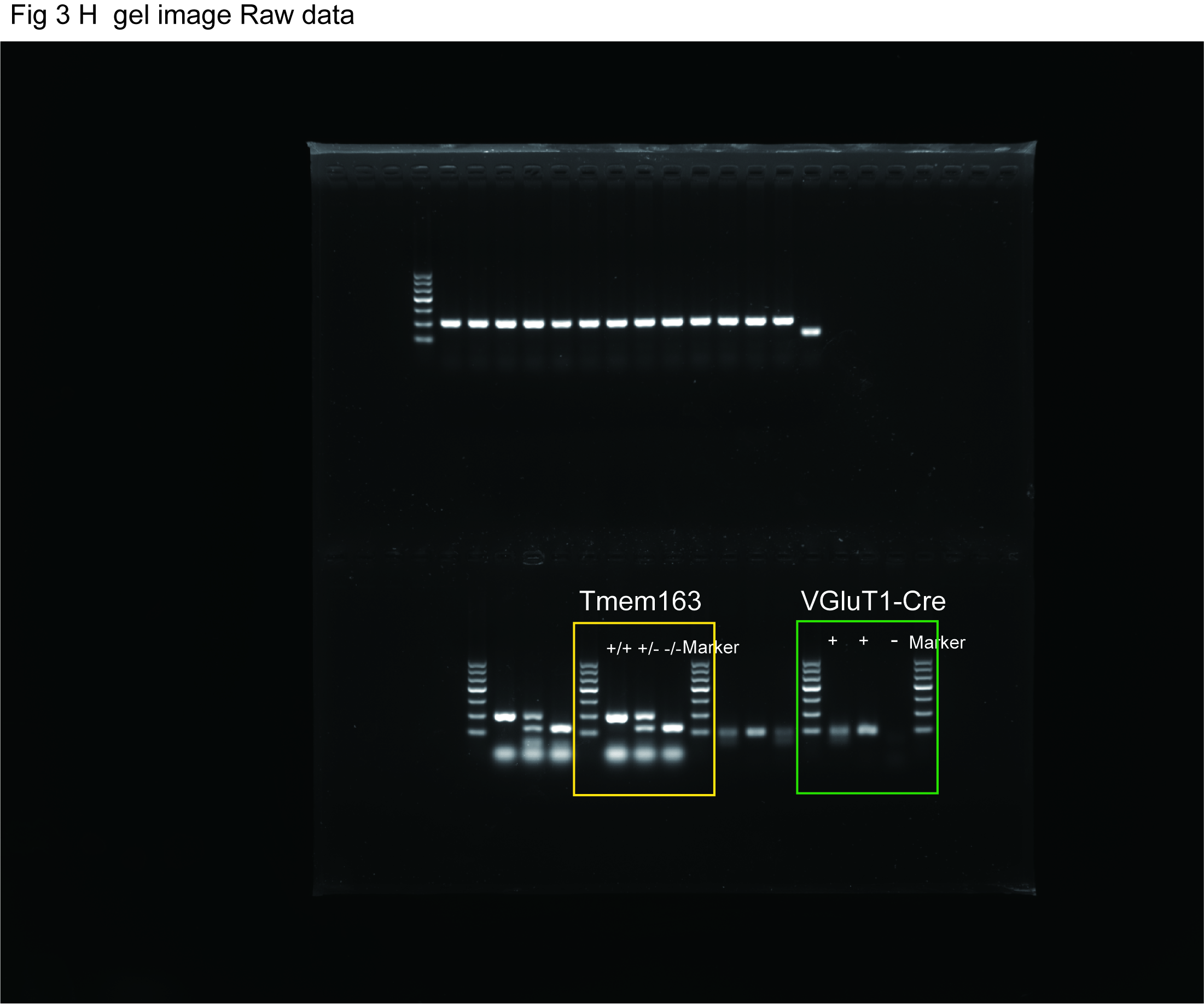

Supplement: S1 Raw Images — (TIF) [file pbio.3002888.s012.tif]
