# Supplementary material for: Elevated vesicular Zn2+ in dorsal root ganglion neurons expressing the transporter TMEM163 causes age-associated itchy skin in mice
Source: PLoS Biol. 2024 Nov 27;22(11):e3002888. doi: 10.1371/journal.pbio.3002888 (PMC11602076; doi:10.1371/journal.pbio.3002888)
Supplement: S1 Table — (DOCX) [file pbio.3002888.s011.docx]

| **Gene name** | **Primer name** | **Primer sequence** | **Product size (bp)** |
| --- | --- | --- | --- |
| ***Slc30a1*** | mZnT1-F | 5'-AACACCAGCAATTCCAACGG-3' | 215 |
|  | mZnT1-R | 5'-GCATTCACGACCACGATCAC |  |
| ***Slc30a2*** | mZnT2-F | 5'-GCTTTTGCACACGACGC | 241 |
|  | mZnT2-R | 5'-CACAGAGATCCTAAGAAGGACCG |  |
| ***Slc30a3*** | mZnT3-F | 5'-CCACCGACGGCTTTCTTTTC | 221 |
|  | mZnT3-R | 5'-GGCCAAGCTGTGTGCTAAAT |  |
| ***Slc30a4*** | mZnT4-F | 5'-TCGGAGAGCTTGTAGAGGTT | 235 |
|  | mZnT4-R | 5'-GAAGGCAGGGAGTGGGAATG |  |
| ***Slc30a5*** | mZnT5-F | 5'-AGGACCTTGCTGCTGTTTGA | 248 |
|  | mZnT5-R | 5'-TCTGCCACCCCTAAAAAGGC |  |
| ***Slc30a6*** | mZnT6-F | 5'-TGGCCTTATCCCAGGACTCA | 230 |
|  | mZnT6-R | 5'-GAGGTGGTGTTGTCTGGAGG |  |
| ***Slc30a7*** | mZnT7-F | 5'-CCATCCTTCAAAGCAACGGC | 265 |
|  | mZnT7-R | 5'-AGCACTGGGGAAGCGTATTT |  |
| ***Slc30a8*** | mZnT8-F | 5'-TGCGAAGACCCTCAGGACTA | 211 |
|  | mZnT8-R | 5'-CCAAGAAGCCGACAAATCCC |  |
| ***Slc30a9*** | mZnT9-F | 5'-AGGATGTTTCCGGGCTTGG | 286 |
|  | mZnT9-R | 5'-GGACTGCTTTGACTGTTTGTGT |  |
| ***Slc30a10*** | mZnT10-F | 5'-CTGCTCTCGGACTCGTTCAA | 191 |
|  | mZnT10-R | 5'-GCCTCCACGAAGATGGTGAA |  |
| ***Tmem163*** | mTmem163-F | 5'-ACTCCTCATCGACATGGTGC | 273 |
|  | mTmem163-R | 5'-CATGTTTGATGGTGGGCGAC |  |
| ***Tmem163* Genotyping** | F1 | 5’-AGGGCCCTTATATATCTCACTGTAA-3’ | MT:202  WT:132 |
|  | R1 | 5’-AAAACCACCTGATGTTGAACTGTG-3’ |  |

Supplemental table：primer list
